# Gametocyte carriage in uncomplicated *Plasmodium falciparum* malaria following treatment with artemisinin combination therapy: a systematic review and meta-analysis of individual patient data

**DOI:** 10.1186/s12916-016-0621-7

**Published:** 2016-05-24

**Authors:** WWARN Gametocyte Study Group

**Affiliations:** Department of Medical Microbiology 268, Radboud University Medical Center, PO Box 9101, 6500HB Nijmegen, the Netherlands; WorldWide Antimalarial Resistance Network (WWARN), Centre for Tropical Medicine and Global Health, Churchill Hospital, CCVTM, University of Oxford, Old Road, Oxford, OX3 7LE UK

**Keywords:** Malaria, *Plasmodium falciparum*, Drug resistance, Gametocyte

## Abstract

**Background:**

Gametocytes are responsible for transmission of malaria from human to mosquito. Artemisinin combination therapy (ACT) reduces post-treatment gametocyte carriage, dependent upon host, parasite and pharmacodynamic factors. The gametocytocidal properties of antimalarial drugs are important for malaria elimination efforts. An individual patient clinical data meta-analysis was undertaken to identify the determinants of gametocyte carriage and the comparative effects of four ACTs: artemether-lumefantrine (AL), artesunate/amodiaquine (AS-AQ), artesunate/mefloquine (AS-MQ), and dihydroartemisinin-piperaquine (DP).

**Methods:**

Factors associated with gametocytaemia prior to, and following, ACT treatment were identified in multivariable logistic or Cox regression analysis with random effects. All relevant studies were identified through a systematic review of PubMed. Risk of bias was evaluated based on study design, methodology, and missing data.

**Results:**

The systematic review identified 169 published and 9 unpublished studies, 126 of which were shared with the WorldWide Antimalarial Resistance Network (WWARN) and 121 trials including 48,840 patients were included in the analysis. Prevalence of gametocytaemia by microscopy at enrolment was 12.1 % (5887/48,589), and increased with decreasing age, decreasing asexual parasite density and decreasing haemoglobin concentration, and was higher in patients without fever at presentation. After ACT treatment, gametocytaemia appeared in 1.9 % (95 % CI, 1.7–2.1) of patients. The appearance of gametocytaemia was lowest after AS-MQ and AL and significantly higher after DP (adjusted hazard ratio (AHR), 2.03; 95 % CI, 1.24–3.12; *P* = 0.005 compared to AL) and AS-AQ fixed dose combination (FDC) (AHR, 4.01; 95 % CI, 2.40–6.72; *P* < 0.001 compared to AL). Among individuals who had gametocytaemia before treatment, gametocytaemia clearance was significantly faster with AS-MQ (AHR, 1.26; 95 % CI, 1.00–1.60; *P* = 0.054) and slower with DP (AHR, 0.74; 95 % CI, 0.63–0.88; *P* = 0.001) compared to AL. Both recrudescent (adjusted odds ratio (AOR), 9.05; 95 % CI, 3.74–21.90; *P* < 0.001) and new (AOR, 3.03; 95 % CI, 1.66–5.54; *P* < 0.001) infections with asexual-stage parasites were strongly associated with development of gametocytaemia after day 7.

**Conclusions:**

AS-MQ and AL are more effective than DP and AS-AQ FDC in preventing gametocytaemia shortly after treatment, suggesting that the non-artemisinin partner drug or the timing of artemisinin dosing are important determinants of post-treatment gametocyte dynamics.

**Electronic supplementary material:**

The online version of this article (doi:10.1186/s12916-016-0621-7) contains supplementary material, which is available to authorized users.

## Background

Malaria remains a leading cause of morbidity and mortality in endemic countries, with an estimated 584,000 deaths and 198 million clinical cases of malaria globally in 2013 [1]. Considerable progress has been made in the last decade in reducing the burden of malaria by wide-scale deployment of insecticide-treated nets and efficacious artemisinin combination therapy (ACT) as first-line antimalarial treatment [2]. To maintain these gains and further move towards malaria elimination, a specific focus on malaria reducing interventions is needed [3]. The transmission of malaria to mosquitoes depends on mature sexual stage parasites, gametocytes, in the human peripheral blood. *Plasmodium falciparum* gametocytaemia has been associated with asexual parasite densities, the duration of malaria symptoms, anaemia and immunity [4, 5]. A large fraction of gametocyte-positive individuals are asymptomatic and the contribution of this asymptomatic reservoir to onward malaria transmission is considerable in many endemic settings [6]. As a consequence, efforts to reduce malaria transmission by antimalarial treatment depend for a large extent on the proportion of malaria-infected individuals that receive treatment [7]. Upon initiation of treatment, gametocytes may persist for several weeks after the clearance of asexual parasites with their longevity and infectivity depending on the treatment dispensed [8, 9], dosing [10] and host immunity [5].

ACT is now recommended universally for the treatment of uncomplicated falciparum malaria. Artemisinins are highly effective against the pathogenic asexual parasite stages [11] and immature gametocytes [12, 13], resulting in a substantial reduction of post-treatment malaria transmission compared to non-artemisinin drugs [9, 14, 15]. The wide-scale deployment of ACTs has been associated with substantial reductions in disease burden across a range of endemic settings [16, 17]. Nevertheless, the transmission reducing effects of ACT may be incomplete because of limited efficacy of artemisinins against mature gametocytes, permitting residual transmission in the first weeks after treatment [9, 15]. Moreover, differences in artemisinin dosing, timing and partner drugs affect their gametocytocidal properties [18, 19].

Because gametocytes are only detected in a fraction of patients by microscopy, individual trials are often insufficiently powered to compare gametocytocidal properties between ACTs or disentangle host and parasite factors that influence gametocyte dynamics. To address this, a pooled analysis of individual-level patient data was undertaken in patients before and after treatment with artemether-lumefantrine (AL), artesunate-amodiaquine (AS-AQ), artesunate-mefloquine (AS-MQ), and dihydroartemisinin-piperaquine (DP).

## Methods

### Data pooling

A search was conducted in PubMed in September 2014 to identify all antimalarial clinical trials published between 1990 and 2014, in which gametocytes were recorded using the search strategy described in the legend of Additional file 1: Table S1. Those who had contributed studies previously to the WorldWide Antimalarial Resistance Network (WWARN) data repository were also invited to participate and asked whether they were aware of any unpublished or ongoing clinical trials involving ACTs, and these additional unpublished studies were also requested. Investigators were invited to participate in this pooled analysis if their studies included (1) uncomplicated *P. falciparum* malaria (alone or mixed infection with another species); (2) asexual parasite quantification at enrolment; (3) gametocyte quantification or prevalence at enrolment; (4) well described methodology for quantifying asexual parasites and gametocytes; and (5) haemoglobin (or haematocrit) estimation at enrolment.

Individual study protocols were available for all trials included, either from the publication or as a metafile submitted with the raw data. Individual patient data from eligible studies were shared, collated and standardised using a previously described methodology [10, 20]. Study reports generated from the formatted datasets were sent back to investigators for validation or clarification. All parasite data were based on microscopic observations.

### Statistical analysis

Statistical analyses were carried out using STATA (Version 13.1) according to an a priori Statistical Analysis Plan [20]. Briefly, we determined: (1) prevalence of gametocytaemia at enrolment (regardless of subsequent treatment regimen); (2) risk of gametocytaemia in patients presenting with no gametocytaemia on enrolment; and (3) time to clearance of gametocytaemia in patients presenting with gametocytaemia. For the comparison of ACT regimens, the analysis was restricted to individuals with no recurrent asexual parasitaemia recorded during follow-up. Multivariable models with random effects were fitted to adjust for study and site heterogeneity: logistic for outcome (1) and Cox regression (with shared frailty) for outcomes (2) and (3). The effect of the following baseline covariates was examined: age, sex, log asexual parasite density, hyperparasitaemia (asexual parasitaemia > 200,000 parasites per μL), haemoglobin/haematocrit, anaemia (haemoglobin concentration < 10 g/dL), presence of/history of fever, nutritional status (based on weight-for-age z-scores in children < 5 years of age), treatment dose of artemisinin derivative, geographic region and malaria transmission intensity [21]. Indicators of parasite clearance time included asexual parasite prevalence and log asexual parasite density on days 1, 2, 3 and the area under the curve of asexual parasite density during days 0–3. Fractional polynomials [22] were used to define the nonlinear relationship between age, haemoglobin concentration and asexual parasite density and the risk of gametocytaemia; to maintain stability, these models were fitted to data from patients ≤ 70 years of age, with haemoglobin between 5 and 18 g/dL and with 500–200,000 asexual parasites per μL. Target dosing for the artemisinin components of the ACTs was defined according to WHO guidelines: ≥ 8.4 mg/kg for AL and ≥ 6 mg/kg for AS-AQ, AS-MQ and DP [23].

Gametocyte carriage at any time after treatment in patients with no recurrent parasitaemia, patients with recrudescent infections and patients with reinfections were compared using multilevel logistic regression models with random effects for study site and subject.

Methods to detect gametocytes by microscopy differed between trials. The sensitivity of microscopy methods was included in the analyses, by classifying studies into one of four categories, as follows: (1) studies in which slides were specifically read for gametocytes, reviewing at least 100 microscopic high power fields or against ≥ 1000 white blood cells (WBC) (4 studies); (2) microscopists specifically instructed to record gametocytes but slides were primarily read for asexual parasites; ≥ 100 microscopic high power fields per ≥ 1000 WBC were read (26 studies); (3) microscopists were specifically instructed to record gametocytes; 50–99 microscopic high power fields per 500–999 WBC were read (33 studies); (4) microscopists were not specifically instructed to record gametocytes or the number of examined high power fields was < 50 or the number of WBC was < 500 (40 studies). For 18 studies, the information on the sensitivity of the microscopy was not available.

Risk of bias within studies was assessed based on (1) study design (randomization, sequence generation, blinding); (2) methodology for gametocyte detection; and (3) the number and proportion of patients with (a) missing outcomes and (b) missing baseline covariates (age, weight, parasitaemia, temperature, haemoglobin/haematocrit). For the final models, two sets of sensitivity analyses were performed. Firstly, a model was refitted with each study’s data excluded, one at a time, and a coefficient of variation around the parameter estimates calculated. This would identify any influential studies, that is, studies with unusual results (due to variations in methodology, patient population, or other reasons) that affect the overall pooled analysis findings. Secondly, for the outcome measure time to gametocytaemia, the impact of incomplete gametocyte carriage data was investigated by refitting the final multivariable model in a subset of patients with complete weekly data for 28 days.

### Ethical approval

All data included in this analysis were obtained in accordance with the laws and ethical approvals applicable to the countries in which the studies were conducted, and were obtained with the knowledge and consent of the individual to which they relate. Data were fully anonymised either before or during the process of uploading to the WWARN repository. Ethical approval to conduct individual participant data pooled analyses was granted to WWARN by the Oxford Tropical Research Ethics Committee (OXTREC-48-09).

## Results

### Characteristics of included studies

**Fig. 1:**
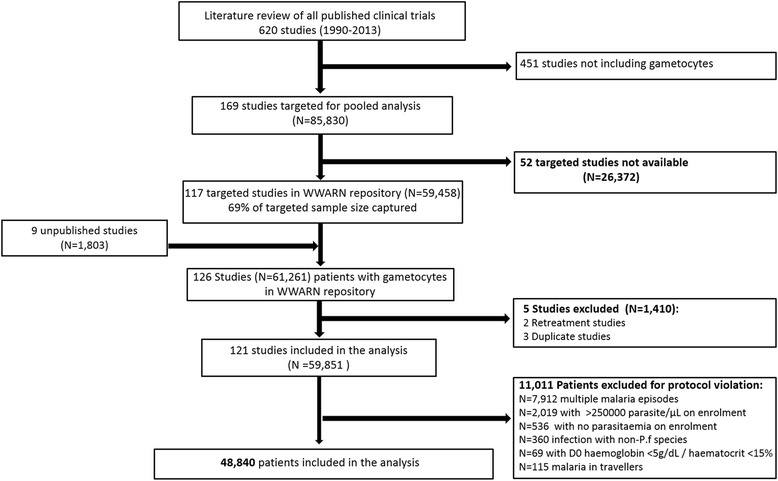
Study profile

In total, 169 published clinical trials were identified that recorded *P. falciparum* gametocytes at enrolment or during follow-up. Investigators of 117 clinical trials (59,458 patients) agreed to contribute their data. In addition, nine unpublished studies (1,803 patients) were shared, one of which was published subsequently. After exclusion of duplicate studies, studies in returning travellers, multiple infection episodes and participants with protocol violations, 48,840 study participants from 121 individual clinical trials were retained (Fig. 1; full list of studies in Additional file 1: Table S1).

### Baseline characteristics

The majority of participants were from Africa (34,377; 70.4 %) or Asia (13,546; 27.7 %) with a minority coming from South America (917; 1.9 %) (Table 1). Most studies involved treatment with an ACT (68.3 % of all participants (33,356/48,840)) with AL being the most commonly used regimen (27.1 %; 13,217/48,840) (Table 2). AS-AQ was given to 17.4 % (8488/48,840) of participants; 50.4 % (4278/8488) of these received a fixed dose combination (FDC), others received a non-fixed dose combination (42.9 %; 3637/8488) or co-blistered AS and AQ (6.8 %; 573/8488). The analyses for AS-AQ were restricted to the FDC regimen (AS-AQ FDC). AS-MQ was administered to 10.6 % (5198/48,840) of participants, in most of the patients (88.1 %; 4580/5198) as a loose combination. The following proportions of patients received less than the recommended dose, AL: 8.3 % (1088/13,086); AS-AQ FDC: 0.1 % (2/4262); AS-MQ: 0.8 % (38/4769) DP: 23.6 % (1488/6315).

**Table 1:** Demographic and baseline characteristics

	Africa		Asia		South America	
	n evaluated	n (%) or median (Range)	n evaluated	n (%) or median (Range)	n evaluated	n (%) or median (Range)
Age						
< 1 year	34361	2502 (7)	13545	60 (0)	915	0 (0)
1–4 years	34361	20473 (60)	13545	1377 (10)	915	0 (0)
5–11 years	34361	6775 (20)	13545	3601 (27)	915	111 (12)
≥ 12 years	34361	4611 (13)	13545	8507 (63)	915	804 (88)
Age (years)	34361	3.3 (0–86.7)	13545	15.0 (0–88.0)	915	23.0 (5.0 – 65.0)
Haemoglobin (g/dL)	24771	9.9 (5.0–19.7)	3139	11.1 (5.0–20)	603	12.2 (7.0–17.3)
Haematocrit (%)	5938	32.8 (15.0–49.8)	8076	36.0 (15.0–50.0)	604	37.0 (18.0 – 50.0)
Derived haemoglobin (g/dL)	26806	9.9 (3.6–19.7)	10937	11.6 (3.6–20)	606	12.2 (7.0–17.3)
Anaemia	26806	13313 (50)	10937	3882 (26)	606	48 (8)
Temperature (°C)	33776	37.9 (34.0–41.5)	10828	37.7 (34.0–42.0)	914	37.5 (35.1 – 42.0)
Fever	34199	21213 (62)	10981	6862 (53)	914	438 (48)
History of fever	7244	6826 (94)	2515	2291 (91)	0	. (.)
Parasitaemia (/μL)	34376	20560 (2–250000)	13546	9720 (0–249818)	915	4514 (0–149925)
Hyperparasitaemia	34376	3223 (9)	13546	1908 (14)	915	3 (0)
Mixed infection	34377	0 (0)	13546	903 (7)	917	0 (0)
Sex (male)	33411	17223 (52)	13243	8015 (61)	917	566 (62)
Weight-for-age z-score						
< 5 years	21765	–0.89 (–5.93 to 4.69)	1403	–1.58 (–5.88 to 4.71)	0	–
< 1 year	2323	–0.68 (–5.93 to 4.69)	56	–0.67 (–4.45 to 4.71)	0	–
1–2 years	9708	–0.96 (–5.91 to 4.54)	414	–1.61 (–5.53 to 4.37)	0	–
3–4 years	8305	–0.99 (–5.3 to 4.38)	869	–1.69 (–5.88 to 2.62)	0	–
Underweight						
< 5 years	21765	3918 (18)	1403	517 (37)	0	–
< 1 year	2323	373 (16)	56	8 (14)	0	–
1–2 years	9708	1846 (19)	414	150 (36)	0	–
3–4 years	8305	1503 (18)	869	338 (39)	0	–
Transmission intensity						
Low	34105	10063 (30)	13246	11884 (90)	917	917 (100)
Moderate	34105	10659 (31)	13246	1362 (10)	917	0 (0)
High	34105	13383 (39)	13246	0 (0)	917	0 (0)

Derived haemoglobin, conversion from haematocrit: haemoglobin = (haematocrit–5.62)/2.60 [40]; Anaemia, haemoglobin < 10 g/dL; Fever, temperature > 37.5 °C; Hyperparasitaemia, parasitaemia > 100,000 parasites per μL; Weight-for-age z-score, calculated using “igrowup” package developed by WHO [41] in children < 5 years of age; Underweight, weight-for-age z-scores < –2

**Table 2:** Overview of treatment, artemisinin combination treatment dosing and formulation

	Treatment		Dosing			
	n evaluated	N (%)	n evaluated	Partner drug dose median (Range)	Artemisinin derivative dose median (Range)	Underdosed n (%)
AL	48840	13217 (27 %)	13086	68.6 (8.9–144.0)	11.4 (1.5–24.0)	1008 (8.3 %)
AS-AQ	48840	8488 (17 %)	8395	31.9 (10.0–91.8)	12.4 (4.0–52.6)	
AS-AQ formulation:						
Co-blistered nFDC	8488	573 (7 %)	573	37.4 (14.8–91.8)	13.5 (4.8–30.0)	
FDC	8488	4278 (50 %)	4262	32.4 (14.5–81.0)	12.0 (5.4–30.0)	2 (0.1 %)
nFDC	8488	3637 (43 %)	3560	30.1 (10.0–60.0)	12.5 (4.0–52.6)	
AS-MQ	48840	5198 (11 %)	4535	25.0 (4.2–85.0)	12.0 (2.3–62.1)	38 (0.8 %)
DP	48840	6453 (13 %)	6315	53.3 (14.5–182.9)	6.7 (1.8–22.9)	1488 (23.6 %)
Other, including non-ACT	48840	15484 (32 %)				

AL, Artemether-Lumefantrine; AS-AQ, Artesunate-Amodiaquine; AS-MQ, Artesunate-Mefloquine; DP, Dihydroartemisinin-piperaquine; nFDC, Non-fixed dose combination, FDC, Fixed dose combination; Underdosed defined as ≤ 8.4 mg/kg artemether dose in AL, < 6 mg/kg dose of artesunate or DHA in other regimens [19]

### Determinants of gametocytaemia at enrolment

**Fig. 2:**
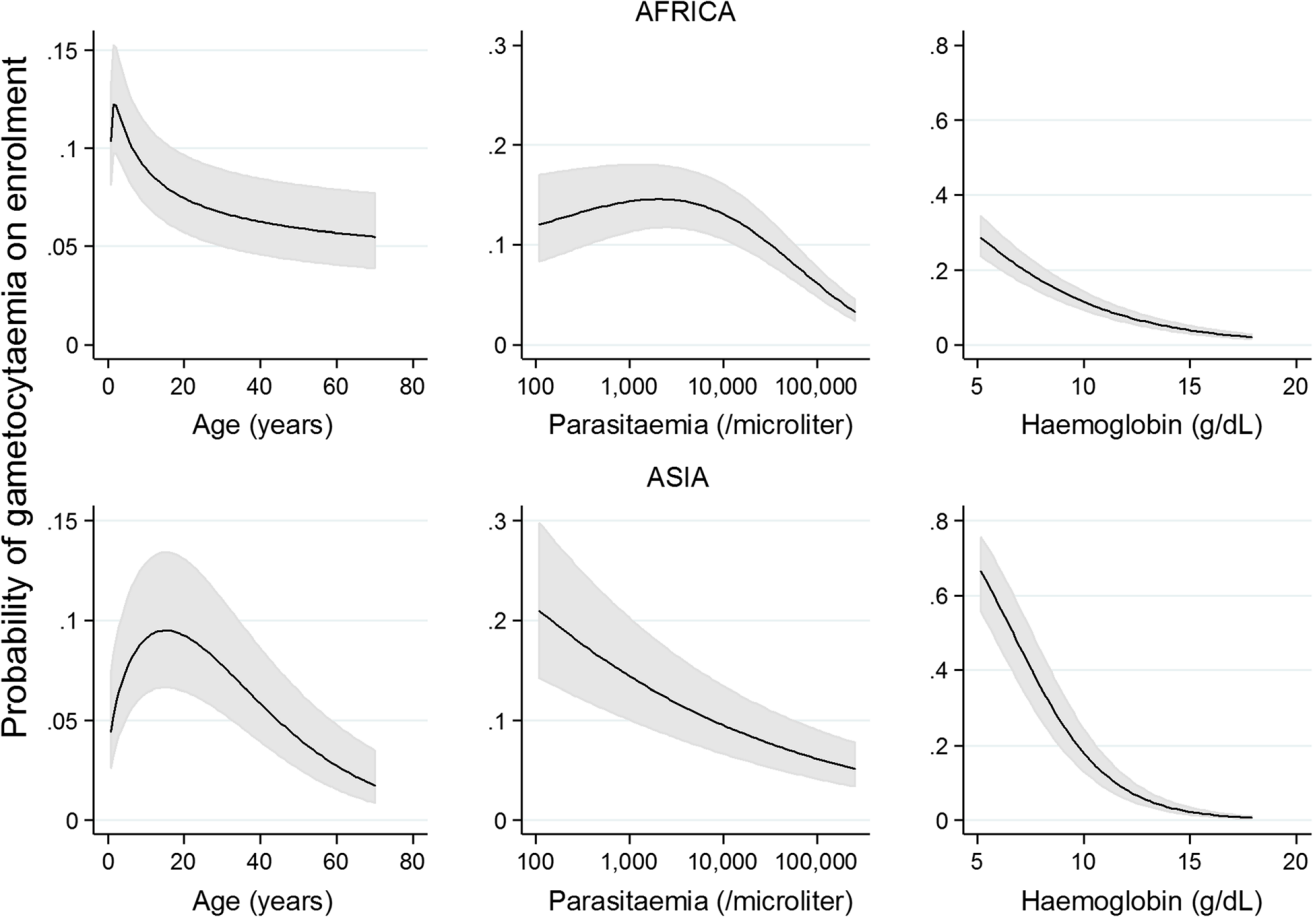
Relationship between gametocytaemia on enrolment and patient age, baseline haemoglobin concentration and asexual parasite density. The predicted probability of gametocyte carriage at enrolment is plotted from the multivariate model; the line indicates the best fit, the shaded area the 95 % confidence interval

Prevalence of gametocytaemia at enrolment was 12.1 % (5887/48,589), and was not significantly influenced by the slide reading method. In Africa, fractional polynomial analysis indicated a gradual decline in the proportion of gametocyte-positive smears with increasing age (Fig. 2); in Asia there was an initial increase in prevalence of gametocytaemia with increasing age in the first 20 years of life, followed by a decline with increasing age thereafter. The differences between African and Asian sites in the association between age and prevalence of gametocytaemia remained apparent when the analysis was restricted to studies with the highest sensitivity of gametocyte detection (≥100 high power fields or ≥ 1,000 WBC examined specifically for gametocytes) and when restricted to children below 5 years of age (Additional file 2: Figure S1). Prevalence of gametocytaemia at enrolment was negatively associated with haemoglobin concentration in all three continents (Table 3; Fig. 2).

**Table 3:** Risk factors for gametocyte prevalence at enrolment

	Africa			Asia			South America		
Parameter	Nobs/Npos (%)	OR (95 % CI)	*P* value	Nobs/Npos (%)	OR (95 % CI)	*P* value	Nobs/Npos (%)	OR (95 % CI)	*P* value
Univariable model									
Age									
< 1 year	2492/403 (16.2)	2.506 (1.978–3.174)	<0.001	60/12 (20.0)	2.240 (1.124–4.460)	0.022	0/0		
1–4 years	20419/2799 (13.7)	2.558 (2.082–3.144)	<0.001	1374/297 (21.6)	2.095 (1.777–2.469)	<0.001	0/0		
5–11 years	6715/495 (7.4)	1.523 (1.245–1.864)	<0.001	3587/563 (15.7)	1.535 (1.354–1.740)	<0.001	111/19 (17.1)	0.834 (0.485–1.432)	0.510
12+ years	4571/179 (3.9)	–		8438/977 (11.6)	–		803/139 (17.3)	–	
Age (years)	34197/3876 (11.3)	0.961 (0.952–0.971)	<0.001	13459/1849 (13.7)	0.975 (0.971–0.980)	<0.001	914/158 (17.3)	0.995 (0.983–1.008)	0.453
Derived haemoglobin (g/dL)	26693/3394 (12.7)	0.785 (0.767–0.803)	<0.001	10854/1433 (13.2)	0.664 (0.645–0.683)	<0.001	606/120 (19.8)	0.600 (0.523–0.688)	<0.001
Anaemia									
Yes	13441/2322 (17.3)	2.062 (1.888–2.253)	<0.001	2871/795 (27.7)	4.846 (4.258–5.516)	<0.001	48/22 (45.8)	3.972 (2.162–7.297)	<0.001
No	13252/1072 (8.1)	Reference		7983/638 (8.0)	Reference		558/98 (17.6)	Reference	
Fever									
Yes	21115/1910 (9.1)	0.583 (0.538–0.631)	<0.001	5816/566 (9.7)	0.738 (0.649–0.839)	<0.001	438/54 (12.3)	0.529 (0.367–0.762)	0.001
No	12925/1950 (15.1)	Reference		5107/730 (14.3)	Reference		476/105 (22.1)	Reference	
Sex									
Female	16119/1782 (11.1)	0.976 (0.908–1.048)	0.501	5205/754 (14.5)	1.083 (0.973–1.206)	0.145	350/52 (14.9)	0.756 (0.525–1.089)	0.133
Male	17128/1963 (11.5)	Reference		7952/1085 (13.6)	Reference		566/107 (18.9)	Reference	
Log_10_ Parasitaemia (/μL)	34212/3879 (11.3)	0.590 (0.554–0.629)	<0.001	13442/1833 (13.6)	0.726 (0.677–0.779)	<0.001	914/158 (17.3)	0.333 (0.212–0.524)	<0.001
Hyperparasitaemia									
Yes	3217/160 (5.0)	0.389 (0.328–0.460)	<0.001	1892/289 (15.3)	0.441 (0.338–0.575)	<0.001	0/0		
No	30995/3719 (12.0)	Reference		12568/1743 (13.9)	Reference		912/158 (17.3)		
Mixed infection									
Yes				892/106 (11.9)	1.112 (0.880–1.404)	0.374			
No				13496/1817 (13.5)	Reference				
Weight-for-age z-score	21701/2996 (13.8)	0.932 (0.901–0.966)	<0.001	1403/305 (21.7)	0.815 (0.723–0.919)	0.001	0/0		
Underweight									
Yes	3904/651 (16.7)	1.234 (1.113–1.368)	<0.001	517/144 (27.9)	1.404 (1.029–1.915)	0.032	0/0		
No	17797/2345 (13.2)	Reference		886/161 (18.2)	Reference		0/0		
TIA									
Low	9995/802 (8.0)	0.990 (0.674–1.454)	0.959	11799/1383 (11.7)	0.251 (0.074–0.850)	0.026	871/147 (16.9)		
Moderate	10575/1069 (10.1)	1.074 (0.801–1.440)	0.631	1361/438 (32.2)	Reference		0/0		
High	13371/2000 (15.0)	Reference		0/0			0/0		
Multivariable model	26669 / 3389 (12.7)			8919 / 929 (10.4)			605 /120 (19.8)		
Age (years)		0.984 (0.974–0.994)	0.001		0.988 (0.982–0.994)	<0.001			
Derived haemoglobin (g/dL)		0.788 (0.770–0.807)	<0.001		0.672 (0.648–0.697)	<0.001		0.581 (0.502–0.672)	<0.001
Log_10_ Parasitaemia (/μL)		0.617 (0.575–0.662)	<0.001		0.735 (0.669–0.807)	<0.001		0.330 (0.184–0.592)	<0.001
Fever		0.633 (0.579–0.691)	<0.001		0.811 (0.689–0.954)	0.011			
Sex (M)					1.252 (1.073–1.462)	0.004		2.144 (1.331–3.454)	0.002

Logistic univariable and multivariable mixed effects analysis by region with presence of gametocytaemia at enrolment as dependent variable. Nobs, number of observations; Npos, number of positive observations; Weight-for-age z-score, calculated using “igrowup” package developed by WHO [41] in children < 5 years of age; Underweight, weight-for-age z-scores < –2; TIA, Transmission intensity areas; Derived haemoglobin, conversion from haematocrit: haemoglobin = (haematocrit–5.62)/2.60 [40]; Anaemia, haemoglobin < 10 g/dL; Fever, temperature > 37.5 °C; Hyperparasitaemia, parasitaemia > 100,000 parasites per μL

In Asia, there was a gradual decline in prevalence of gametocytaemia with increasing asexual parasite density across the entire range of asexual parasite densities that were observed (Fig. 2). In Africa, when asexual parasite density exceeded 10,000 parasites/μL, there was a gradual decline in prevalence of gametocytaemia with increasing asexual parasite density. At lower parasite densities the uncertainty around estimates was larger and the association between prevalence of gametocytaemia and the logarithm of asexual parasite density was non-linear (Fig. 2). These differences between African and Asian sites remained apparent when the analysis was restricted to studies with the highest sensitivity of gametocyte detection (Additional file 2: Figure S1).

In all regions, individuals presenting with fever (axillary temperature >37.5 °C or reporting of febrile symptoms) were less likely to present with gametocytaemia and this remained significant after adjusting for covariates in both African (adjusted odds ratio (AOR), 0.63; 95 % CI, 0.58–0.69; *P* < 0.001) and Asian (AOR, 0.81; 95 % CI, 0.69–0.95; *P* = 0.011) patients. Male gender was a predictor of prevalence of gametocytaemia at enrolment in studies in Asia (AOR, 1.25; 95 % CI, 1.07–1.46; *P* = 0.004) and South America (AOR, 2.14; 95 % CI, 1.33–3.45; *P* = 0.002) but not Africa (Table 3). Children under 5 years of age who were malnourished (weight-for-age z-scores < –2) had a higher prevalence of gametocytaemia at enrolment compared to well-nourished children in Africa (OR, 1.23; 95 % CI, 1.11–1.37; *P* < 0.001) and in Asia (OR, 1.40; 95 % CI, 1.03–1.92; *P* = 0.032) but this was not significant in the multivariable analysis (Additional file 3: Table S2).

### Gametocytaemia after artemisinin combination therapy

#### No gametocytaemia at enrolment

**Fig. 3:**
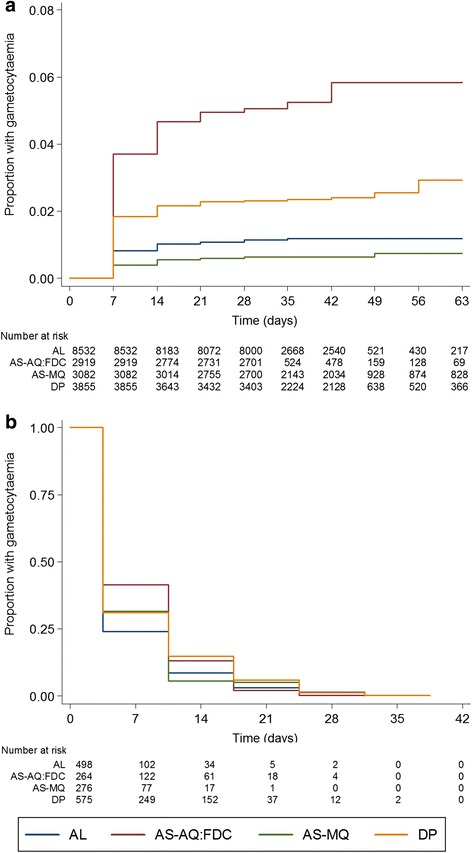
Gametocyte carriage by artemisinin-combination therapy. **a** Development of gametocytaemia after treatment with artemether-lumefantrine (AL), artesunate-amodiaquine fixed-dose combination (AS-AQ-FDC), artesunate-mefloquine (AS-MQ) or dihydroartemisinin-piperaquine (DP); evaluated in patients with no gametocytes on enrolment. **b** Gametocyte clearance, adjusted for initial gametocyte count, evaluated in patients with gametocytes on enrolment. Only patients with no recurrent infection recorded were included

Amongst the 18,388 individuals presenting without patent gametocytaemia by microscopy who were treated with an ACT, the Kaplan–Meier estimate of risk of appearance of gametocytaemia within 28 days was 1.9 % (95 % CI, 1.7–2.1) (Fig. 3a). This proportion was similar in African and Asian studies. After controlling for confounding factors, the risk of appearance of gametocytaemia correlated negatively with age, haemoglobin concentration, fever and asexual parasite density at enrolment (Table 4). Appearance of gametocytaemia was lowest after AS-MQ or AL treatment and significantly higher after DP (adjusted hazard ratio (AHR), 2.03; 95 % CI, 1.24–3.32; *P* = 0.005 compared to AL) and AS-AQ (AHR, 4.01; 95 % CI, 2.40–6.72; *P* < 0.001 compared to AL) (Fig. 3a, Table 4). A dose of the artemisinin component < 8 mg/kg was associated with an increased chance of appearance of gametocytaemia after treatment with DP (AHR, 2.78; 95 % CI, 1.18–6.55; *P* = 0.020) but not after treatment with any of the other ACTs (Additional file 4: Table S3). No association was observed for a dose of the artemisinin component < 6 mg/kg either for all treatment combined or for DP alone.

**Table 4:** Factors associated with the development of gametocytaemia after enrolment in individuals without microscopically detected gametocytes before treatment with artemisinin combination therapy

Parameter	Nobs	Npos	per	Hazard ratio (95 % CI)	*P* value
Univariable model					
ACT^a^					
AS-MQ	3082	20	0.6	0.763 (0.392–1.484)	0.425
DP	3855	93	2.4	2.746 (1.773–4.253)	<0.001
AS-AQ: FDC	2919	151	5.2	4.094 (2.540–6.600)	<0.001
AL	8532	97	1.1	Reference	
Age^a^					
< 1 year	776	23	3.0	2.435 (1.268–4.676)	0.008
1–4 years	7772	236	3.0	2.780 (1.698–4.552)	<0.001
5–11 years	4102	58	1.4	1.928 (1.225–3.035)	0.005
12+ years	5735	44	0.8	Reference	
Age (years)	18385	361	2.0	0.965 (0.946–0.984)	<0.001
Derived haemoglobin (g/dL)	14357	295	2.1	0.809 (0.758–0.862)	<0.001
Anaemia					
Yes	5505	183	3.3	1.824 (1.402–2.373)	<0.001
No	8852	112	1.3	Reference	
Fever					
Yes	10569	173	1.6	0.594 (0.470–0.749)	<0.001
No	7244	173	2.4	Reference	
Sex					
Female	8427	160	1.9	0.931 (0.754–1.149)	0.505
Male	9658	196	2.0	Reference	
Hyperparasitaemia					
Yes	1543	19	1.2	0.615 (0.384–0.984)	0.043
No	16845	342	2.0	Reference	
Log_10_ parasitaemia (/μL)	18388	361	2.0	0.731 (0.619–0.864)	<0.001
Weight-for-age score					
Underweight^b^	8341	257	3.1	0.825 (0.744–0.915)	<0.001
Yes	1418	80	5.6	1.768 (1.343–2.326)	<0.001
No	6923	177	2.6	Reference	
Region					
Asia	3895	55	1.4	1.078 (0.356–3.263)	0.894
South America	615	8	1.3	0.482 (0.035–6.564)	0.584
Africa	13878	298	2.1	Reference	
TIA^a^					
Low	8406	64	0.8	0.371 (0.159–0.866)	0.022
Moderate	5120	129	2.5	0.746 (0.426–1.306)	0.305
High	4449	161	3.6	Reference	
Multivariable model	14051	291	2.1		
ACT:					
AS-MQ				0.566 ( 0.225–1.420)	0.225
DP				2.029 (1.240–3.317)	0.005
AS-AQ: FDC				4.014 (2.398–6.719)	<0.001
AL				Reference	
Age					
< 1 year				1.707 ( 0.778–3.747)	0.269
1–4 years				2.303 (1.208–4.392)	0.011
5–11 years				1.418 (0.795–2.527)	0.237
12+ years				Reference	
Derived haemoglobin (g/dL)				0.828 (0.774–0.886)	<0.001
Fever				0.653 (0.503–0.848)	0.001
Log_10_ parasitaemia (/μL)				0.757 (0.624–0.917)	0.004

Cox regression mixed effects model for time to gametocytaemia

Nobs, Number of observations; Npos, Number of positive observations; Weight-for-age z-score, calculated using “igrowup” package developed by WHO [41] in children < 5 years of age; Underweight, weight-for-age z-scores < –2; TIA, Transmission intensity areas. ^a^ Proportional hazards assumption not satisfied; ^b^ In multivariable analysis: HR, 1.51; 95 % CI, 1.13–2.02; *P* = 0.005, after adjusting for covariates in the main model

#### Gametocytaemia at enrolment

A total of 2433 patients treated with an ACT were gametocytaemic at enrolment and had no recurrent infection. Overall, 57.4 % (95 % CI, 55.4–59.4) of these patients cleared gametocytaemia by day 7, 78.4 % (95 % CI, 76.5–80.2) by day 14 and 88.2 % (95 % CI, 86.6–89.6) by day 21. The only independent determinants of gametocyte clearance were initial gametocyte density (AHR, 0.87; 95 % CI, 0.83–0.91; *P* < 0.001 for log increase in gametocyte density) and the type of ACT given (Additional file 5: Table S4, Fig. 3b). Compared to AL, gametocytaemia clearance was significantly faster with AS-MQ (AHR, 1.26; 95 % CI, 1.00–1.60; *P* = 0.054) and slower with DP (AHR, 0.74; 95 % CI, 0.63–0.88; *P* = 0.001) (Fig. 3b). For the AS-AQ FDC, the rate of gametocytaemia clearance was significantly slower compared to that of AS-MQ (HR, 0.64; 95 % CI, 0.48–0.85; *P* = 0.002), and non-significantly slower compared to AL (HR, 0.80; 95 % CI, 0.63–1.02; *P* = 0.072). The overall observed proportion of patients who cleared gametocytes by day 7 was 64.4 % for AL, 61.7 % for AS-MQ, 52.3 % for DP, and 47.8 % for AS-AQ, while by day 14 gametocytes were cleared by 85.7 %, 90.2 %, 70.3 %, and 72.1 % of patients, respectively.

### Gametocytaemia in relation to asexual parasite clearance time and treatment response

**Fig. 4:**
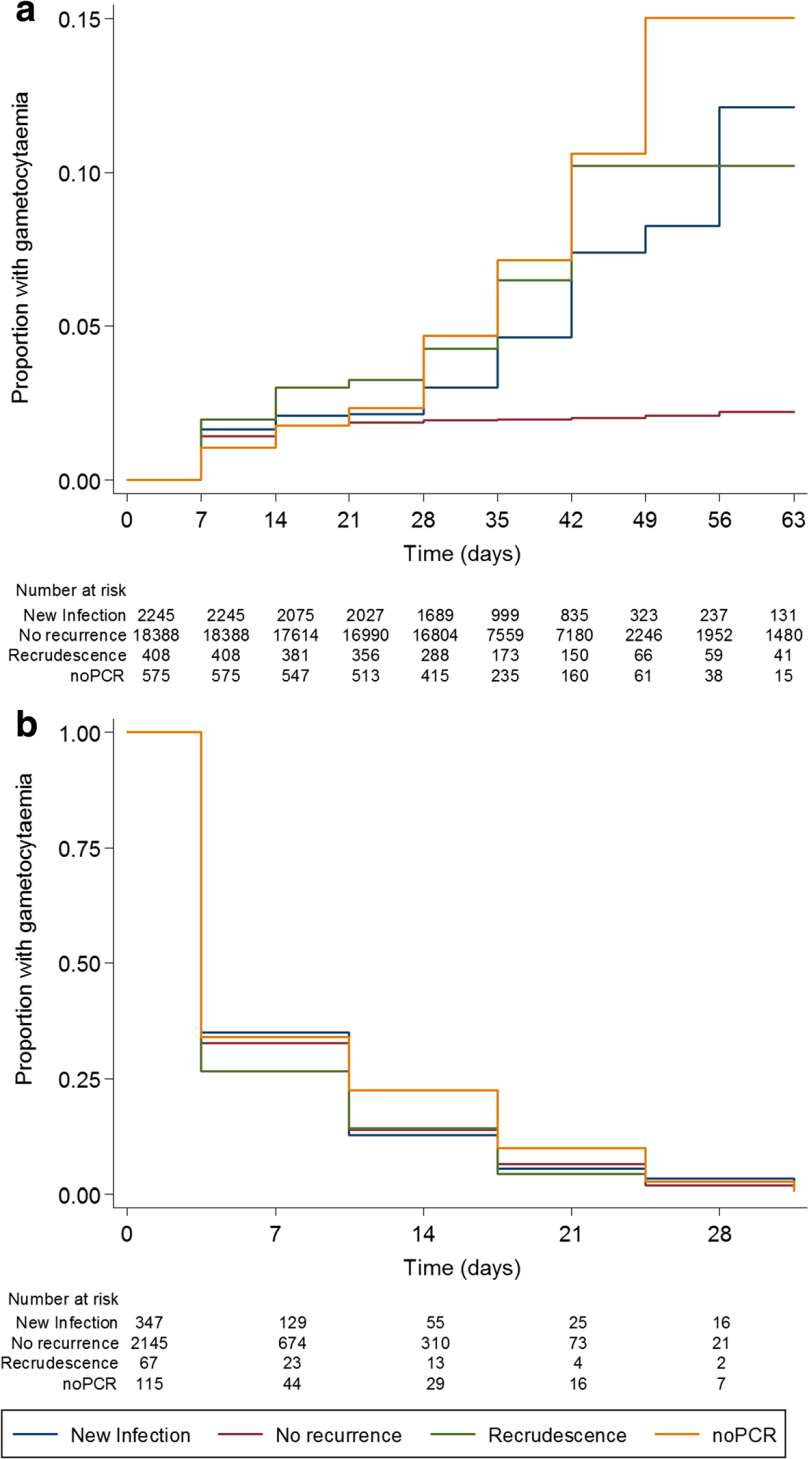
Gametocyte carriage by treatment outcome. **a** Development of gametocytaemia after treatment; evaluated in patients with no gametocytes on enrolment. **b** Clearance of gametocytaemia, adjusted for initial gametocyte count, evaluated in patients with gametocytaemia on enrolment

Asexual parasite clearance was rapid for all treatments with 8.8 %, 9.1 %, 6.4 %, and 7.8 % of patients having residual asexual parasites after 2 days treatment with AL, AS-MQ, AS-AQ-FDC, and DP, respectively. On day 3, these figures were 0.8 %, 1.3 %, 0.4 %, and 0.7 %. Residual asexual parasite prevalence on day 1, 2 or 3 was not associated with gametocytaemia clearance or the appearance of gametocytaemia in univariable or multivariable analysis. Individuals who experienced PCR-confirmed treatment failure by day 28 were more likely to be gametocytaemic on any day during follow-up (AOR, 2.12; 95 % CI, 1.08–4.34; *P* = 0.025) and develop gametocytaemia after day 7 (AOR, 9.05; 95 % CI, 3.74–21.91; *P* < 0.001) compared to patients with no recorded recurrence and at least 28 days follow-up. Similarly, the increased risk of gametocytaemia on any day during follow-up (AOR, 1.95; 95 % CI, 1.37–2.77; *P* < 0.001) and of developing gametocytaemia after day 7 (AOR, 3.03; 95 % CI, 1.66–5.54; *P* < 0.001) was observed in individuals with reinfection (Fig. 4a). This association was not explained by differences in artemisinin dosing. Gametocytaemia clearance in individuals with gametocytaemia prior to treatment was not associated with treatment outcome (Fig. 4b).

### Assessment of potential bias

Attrition bias of the included studies is presented in Additional file 6: Table S5. Although many studies were not blinded, the blinding of the independent outcome laboratory assessments (i.e. microscopy readings to measure gametocytaemia and PCR classification of treatment outcome are performed by laboratory staff not directly involved in the study), minimize the risk of bias in outcome assignment. We consider publication bias unlikely since gametocytaemia measurements were a primary outcome in only 2 (out of 121) publications and gametocytaemia results are unlikely to have influenced the decision to publish. Sensitivity analyses showed that exclusion of any of the studies did not change the main conclusions of the analysis (Additional file 7: Table S6). Results for time to gametocytaemia were also confirmed for all covariates except for age when analysis was restricted to individuals with complete weekly data on gametocytaemia (Additional file 8: Table S7 and Additional file 9: Figure S2). The fact that the effect of age was lost may be due to a considerable loss of observations in this sub-analysis that differed by age groups: 12 %, 15 %, 33 %, and 32 % of patients in groups <1 year, 1–4 years, 5–11 years, and ≥12 years of age were not included in the sub-analysis.

## Discussion

We analysed data from nearly 50,000 patients from trials that included measures of gametocytaemia by blood smears. The prevalence of gametocytaemia before and after treatment was greatest in young patients, and those with lower asexual parasite density, anaemia and absence of fever. After treatment with an ACT, the appearance and clearance of gametocytaemia was determined by the type of ACT with AL and AS-MQ being most efficacious in preventing post-treatment gametocyte carriage.

Gametocytaemia is essential for onward transmission of malaria infections to mosquitoes. Understanding factors that influence gametocytaemia prior to treatment and the gametocytocidal properties of antimalarial drugs is of great relevance for interventions that aim to reduce malaria transmission. Mature *P. falciparum* gametocytes first appear in the human bloodstream 7 to 15 days after the initial wave of their asexual parasite progenitors. This long maturation process and the impacts of human and parasite factors associated with gametocyte production [5] result in considerable variation in the proportion of malaria patients harbouring gametocytes upon presentation with clinical illness. We observed that the same host characteristics influenced gametocytaemia before and after treatment. The prevalence of gametocytaemia was higher in patients with anaemia and without concurrent fever [4, 24]. Reduced haemoglobin concentrations are often a consequence of prolonged duration of infections or recurrent malaria episodes [25, 26], both of which have been associated with increased gametocyte production [4]. Anaemia may also be an independent predictor of gametocytaemia [4, 27] since low haemoglobin concentrations and reticulocytosis directly stimulate gametocyte production [28, 29]. The association between asexual parasite density at enrolment and gametocytaemia was different in Asian and African settings. In Asian studies, the prevalence of gametocytaemia showed a gradual negative association with asexual parasite density [4], whilst in Africa, this negative association was only apparent at asexual parasite densities above 5,000 parasites/μL. These setting-dependent patterns may explain previous inconsistent reports on the association between asexual parasite densities and gametocytaemia [4, 27, 30, 31]. These three predictors of gametocytaemia (anaemia, lower asexual parasite density and absence of fever) may all reflect chronic infections that, because of their longer duration, may be more likely to present with gametocytaemia. Host immunity and the likelihood of super-infections vary significantly with transmission intensity and both influence asexual parasite densities and gametocyte dynamics. Age is a useful surrogate of immunity. In African studies, there was a gradual decrease in the prevalence of gametocytaemia with increasing age, while in Asia, the prevalence of gametocytaemia increased until approximately 20 years of age followed by a general decline thereafter. Further studies are needed to determine whether this pattern is explained by host-factors or by age or occupation-associated malaria exposure in Asian settings.

Patients presenting with gametocytaemia cleared their gametocytaemia rapidly following ACT, with 57 % of patients being gametocyte-free by day 7 and 88 % by day 21. The rate of gametocytaemia clearance varied significantly with the ACT regimen. Differential effects of ACT on post-treatment gametocytaemia have been reported previously, but with contradicting results [32–34]. Our large meta-analysis revealed that both the appearance and duration of gametocytaemia were 2-fold and 25 % lower, respectively, in AL- compared to DP-treated patients. In individuals treated with DP, a lower treatment dose was associated with an increased appearance of gametocytaemia after treatment. We previously demonstrated that treatment failure is also associated with DP dosing [10] and the World Health Organization recently increased the dose recommendation for DP to ensure a minimum of 7.5 mg/kg total dose of dihydroartemisinin in children < 25 kg [35]. The appearance of gametocytaemia after AS-AQ FDC was markedly more prevalent than after either AL or AS-MQ. Furthermore, gametocytaemia clearance was slower after AS-AQ FDC compared to AS-MQ. This striking difference of AS-AQ FDC compared to AL and AS-MQ could not be explained by differences in total artemisinin dosing or treatment outcome. These differential effects of ACTs may relate to the frequency of artemisinin dosing or to the activity of the non-artemisinin partner drug. In vitro drug screening assays indicate similar activity of lumefantrine and amodiaquine against mature gametocytes [36], whilst developing gametocytes appear more susceptible to mefloquine and lumefantrine than to amodiaquine [37]. This would suggest that the maturation of developing gametocytes after initiation of treatment differs between ACT regimens, and this has consequences for post-treatment gametocytaemia.

Contrary to previous studies [38, 39], we found no association between the rate of asexual parasite clearance and gametocytaemia during follow-up. For chloroquine and sulphadoxine-pyrimethamine treatment, post-treatment gametocytaemia and malaria transmission to mosquitoes have been proposed as early parasitological indicators of reduced drug sensitivity [40, 41]. In our study, >98 % of all patients cleared their infections by day 2 post-initiation of treatment. Patients subsequently failing treatment were at 15-fold greater risk of gametocytaemia than those successfully treated, and this was similar for both PCR confirmed recrudescent and new infections. The timing of gametocytaemia coincided with the recurrent asexual parasitaemia. Since the earliest developmental stages of gametocytes are sequestered for 6–8 days in the bone marrow [42], this suggests that gametocyte production started before reappearing asexual parasites were detected by microscopy. The strong association of gametocytaemia with recrudescent infections and new infections warns against a simplistic comparison of treatment regimens based on gametocytaemia shortly after treatment. Initial treatment efficacy and post-treatment prophylaxis that postpones new infection, and therefore de novo gametocyte production, are important determinants of the impact of ACT regimens on malaria transmission.

Whilst our analysis focuses on peripheral gametocytaemia, it is important to acknowledge that this is a surrogate marker of malaria transmission potential. The infectivity of persisting or appearing gametocytes may be affected by the type of antimalarial treatment [9]. Antimalarial drugs may also influence gametocyte sex-ratio [43], which is an important determinant of transmission success, although there is currently no evidence for a differential effect of ACT regimens on male and female gametocytes. The only available study that directly determined infectiousness to mosquitoes after ACT regimens compared in this study supports our findings, reporting a two-fold higher mosquito infection rate after DP compared to AL [18], which is consistent with our finding of significantly higher risk of gametocyte appearance after DP (AHR, 2.03; 95 % CI, 1.24–3.34; *P* = 0.005 compared to AL). Gametocyte densities commonly fluctuate around the microscopic threshold for detection and the use of molecular gametocyte detection tools would have uncovered a higher proportion of gametocyte carriers [5] at densities capable of contributing to onward malaria transmission [44]. The addition of a single low primaquine dose to ACT can substantially reduce the duration of low density gametocytaemia after treatment [45] and prevent transmission to mosquitoes [46, 47] but primaquine is currently not routinely added to ACTs for treatment of uncomplicated malaria. Importantly, although the gametocytocidal properties of first-line ACTs may influence community-wide transmission [16, 48], this effect may be modest if transmission is largely driven by asymptomatic individuals who do not seek treatment. The inclusion of these asymptomatically infected individuals in treatment campaigns may have a much larger impact on malaria transmission than the choice of ACT for first-line treatment [6, 7].

Our analysis was purposefully restricted to microscopic findings on gametocytaemia, for which most data are available. Although this approach will have missed some gametocyte carriers, this would not affect the comparison of treatment arms. Studies where microscopy, molecular gametocyte data and infectivity results are available indicate that these methods lead to the same conclusions on the comparative effects of antimalarials on post-treatment gametocyte dynamics and infectivity [15, 18].

## Conclusions

In conclusion, we identified independent risk factors for the prevalence of gametocytaemia in patients with uncomplicated falciparum malaria in studies conducted on three continents. AS-MQ and AL are superior ACT options in preventing gametocytes shortly after treatment compared to DP or AS-AQ. We hypothesize that this difference is due to the non-artemisinin partner drug defining post-treatment gametocyte dynamics.

## Additional files

Additional file 1: Table S1. Overview of all included studies. ^1^ The sensitivity of microscopy methods was classified into one of four categories: 1 = studies in which slides were specifically read for gametocytes, reviewing at least 100 microscopic high power fields or against ≥ 1000 white blood cells (WBC); 2 = microscopists specifically instructed to record gametocytes but slides were primarily read for asexual parasites; ≥ 100 microscopic high power fields per ≥ 1000 WBC were read; 3 = microscopists were specifically instructed to record gametocytes; 50–99 microscopic high power fields per 500–999 WBC were read; 4 = microscopists were not specifically instructed to record gametocytes or the number of examined high power fields was < 50 or the number of WBC was < 500. ^2^ All treatment combinations are loose, unless stated. FDC, fixed dose combination. AS, Artesunate; MQ, Mefloquine; AL, Artemether-lumefantrine; DP, Dihydrartemisinin-piperaquine; SP, Sulphadoxine-pyrimethamine; AQcb, AQ co-blisterered loose combination; HL, Halofantrine; QN, Quinine; AM, Artemether, AV, Atovaquone; PG, Proguanil; CQ, Chloroquine; CDA, Chlorproguanil-dapsone-artesunate; Tet, Tetracycline; CL, Clindamycine. Search strategy: Published prospective trials were identified by the application of the key terms ((malaria OR plasmod*) AND (amodiaquine OR atovaquone OR artemisinin OR arteether OR artesunate OR artemether OR artemotil OR azithromycin OR artekin OR chloroquine OR chlorproguanil OR cycloguanil OR clindamycin OR coartem OR dapsone OR dihydroartemisinin OR duo-cotecxin OR doxycycline OR halofantrine OR lumefantrine OR lariam OR malarone OR mefloquine OR naphthoquine OR naphthoquinone OR piperaquine OR primaquine OR proguanil OR pyrimethamine OR pyronaridine OR quinidine OR quinine OR riamet OR sulphadoxine OR tetracycline OR tafenoquine)) though the PubMed library. Studies on prevention, prophylaxis, review, animal studies or patients with severe malaria were excluded. (DOCX 155 kb) Additional file 2: Figure S1. Relationship between gametocytaemia on enrolment and baseline haemoglobin concentration, parasitaemia and patient age. The predicted probability of gametocyte carriage at enrolment is plotted from the multivariate model; the line indicates the best fit, the shaded area the 95 % CI. Only patients from studies with gametocyte detection sensitivity in category 1 or 2 were used for this analysis (≥ 100 high power fields or ≥ 1000 WBC examined specifically for gametocytes). For the analysis on age, only children < 5 years of age were included in the analysis. (TIF 480 kb) Additional file 3: Table S2. Independent risk factors for the prevalence of gametocytaemia at enrolment in children aged 1–5 years. Logistic multivariable analysis by region with prevalence of gametocytaemia at enrolment as dependent variable. Nobs, Number of observations; Npos, Number of positive observations. The relationship between gametocyte prevalence at enrolment and age is statistically significant (*P* < 0.001) although not linear, see Additional file 2: Figure S1. Malnutrition (underweight) was not an independent predictor (AOR, 1.11; 95 % CI, 0.99–1.26; *P* = 0.083) in Africa and (AOR, 0.92; 95 % CI, 0.47–0.83; *P* = 0.823) in Asia, after adjustment for age, haemoglobin, parasitaemia and fever (after polynomial transformations, as presented in Additional file 2: Figure S1). (DOC 28 kb) Additional file 4: Table S3. The effect of treatment dosing on the appearance of gametocytaemia in participants without microscopically detected gametocytaemia before treatment (time to gametocytaemia) and clearance of gametocytaemia in participants with gametocytaemia at enrolment (time to clearance). All analyses of time to clearance are adjusted for log of the initial gametocyte density. Nobs, Number of observations; Npos, Number of positive observations. Under-dosed defined as ≤ 8.4 mg/kg artemether dose in AL, < 6 mg/kg dose of artesunate or DHA in other regimens [19]. In the multivariate model estimates are adjusted for other covariates, for time to gametocytaemia: covariates identified in the full final model presented in Table 4; for time to clearance: ACT, since no other covariates other than ACT were identified in the final model there were no multivariate models fitted within each ACT. ND, No data, HR could not be estimated as there were no patients with gametocytaemia in the under-dose/low-dose group. (DOC 86 kb) Additional file 5: Table S4. Factors associated with the clearance of gametocytaemia after enrolment in individuals who were gametocytaemic before treatment with artemisinin combination therapy. Nobs, Number of observations; N cleared, Number of patients with day of clearance of gametocytaemia recorded. Derived haemoglobin, conversion from haematocrit: haemoglobin = (haematocrit – 5.62)/2.60 [40]; Anaemia, haemoglobin < 10 g/dL; Fever, temperature > 37.5 °C; Hyperparasitaemia, parasitaemia > 100,000 parasites per μL; weight-for-age z-score, calculated using “igrowup” package developed by WHO [41] in children < 5 years of age; Underweight, weight-for-age z-scores < –2. Proportional hazards assumption not satisfied for transmission intensity areas, Region, and artemisinin combination therapy. (DOC 59 kb) Additional file 6: Table S5. Risk of bias in individual studies included in the analysis. ACT, Artemisinin combination therapy. ^1^ For trials with non-ACTs, data were only analysed for gametocytaemia on enrolment and regimens, arms, randomization, concealment of treatment, sequence generation and treatment blinding are given as not applicable (NA). ^2^ Includes exclusions due to study design (i.e. travellers, repeated episodes). ^3^ Evaluated in all patients except for exclusions due to study design or protocol violations. ^4^ Evaluated on all included patients treated with ACT and without gametocytaemia on enrolment. ^5^ Proportion of patients with time to gametocyte data available but incomplete day 28 follow-up. ^6^ Evaluated on all included patients with gametocytaemia on enrolment treated with ACT. ^7^ The sensitivity of microscopy methods was classified into one of four categories: 1 = studies in which slides were specifically read for gametocytes, reviewing at least 100 microscopic high power fields or against ≥ 1000 white blood cells (WBC); 2 = microscopists specifically instructed to record gametocytes but slides were primarily read for asexual parasites ; ≥ 100 microscopic high power fields per ≥1000 WBC were read; 3 = microscopists were specifically instructed to record gametocytes; 50–99 microscopic high power fields per 500–999 WBC were read; 4 = microscopists were not specifically instructed to record gametocytes or the number of examined high power fields was < 50 or the number of WBC was < 500. ^8^No data, no patients with sufficient gametocyte follow-up data that could be included in the analysis. (PDF 408 kb) Additional file 7: Table S6. Factors associated with the development of gametocytaemia after enrolment in individuals who were gametocyte-free before treatment with artemisinin combination therapy. Cox regression model for time to gametocytaemia. Only patients with complete 28-day follow-up are included. (DOC 35 kb) Additional file 8: Table S7. Sensitivity analysis: variation in model coefficients after exclusion of individual studies. ^1^ Estimates as obtained in the final multivariate models and listed in main tables. ^2^ RSD, Relative standard deviation was calculated as a ratio of standard deviation to mean of the estimates (odds ratio or hazard ratio) calculated by fitting models with one study excluded at a time. (DOC 44 kb) Additional file 9: Figure S2. Development of gametocytaemia after treatment evaluated in patients with no gametocytaemia on enrolment and full 28-day follow-up. A: Development of gametocytaemia by artemisinin combination therapy. B: Development of gametocytaemia by treatment outcome. (TIF 284 kb)
