# Supplementary material for: Gametocyte carriage in uncomplicated Plasmodium falciparum malaria following treatment with artemisinin combination therapy: a systematic review and meta-analysis of individual patient data
Source: BMC Med. 2016 May 24;14:79. doi: 10.1186/s12916-016-0621-7 (PMC4879753; doi:10.1186/s12916-016-0621-7)
Supplement: Additional file 1: Table S1. — Overview of all included studies. 1 The sensitivity of microscopy methods was classified into one of four categories: 1 = studies in which slides were specifically read for gametocytes, reviewing at least 100 microscopic high power fields or against ≥ 1000 white blood cells (WBC); 2 = microscopists specifically instructed to record gametocytes but slides were primarily read for asexual parasites; ≥ 100 microscopic high power fields per ≥ 1000 WBC were read; 3 = microscopists were specifically instructed to record gametocytes; 50–99 microscopic high power fields per 500–999 WBC were read; 4 = microscopists were not specifically instructed to record gametocytes or the number of examined high power fields was < 50 or the number of WBC was < 500. 2 All treatment combinations are loose, unless stated. FDC, fixed dose combination. AS, Artesunate; MQ, Mefloquine; AL, Artemether-lumefantrine; DP, Dihydrartemisinin-piperaquine; SP, Sulphadoxine-pyrimethamine; AQcb, AQ co-blisterered loose combination; HL, Halofantrine; QN, Quinine; AM, Artemether, AV, Atovaquone; PG, Proguanil; CQ, Chloroquine; CDA, Chlorproguanil-dapsone-artesunate; Tet, Tetracycline; CL, Clindamycine. Search strategy: Published prospective trials were identified by the application of the key terms ((malaria OR plasmod*) AND (amodiaquine OR atovaquone OR artemisinin OR arteether OR artesunate OR artemether OR artemotil OR azithromycin OR artekin OR chloroquine OR chlorproguanil OR cycloguanil OR clindamycin OR coartem OR dapsone OR dihydroartemisinin OR duo-cotecxin OR doxycycline OR halofantrine OR lumefantrine OR lariam OR malarone OR mefloquine OR naphthoquine OR naphthoquinone OR piperaquine OR primaquine OR proguanil OR pyrimethamine OR pyronaridine OR quinidine OR quinine OR riamet OR sulphadoxine OR tetracycline OR tafenoquine)) though the PubMed library. Studies on prevention, prophylaxis, review, animal studies or patients with severe malaria were excluded. (DOCX 155 kb) [file 12916_2016_621_MOESM1_ESM.docx]

**Supplementary Table S1. Overview of all included studies.**

| **Study ID** | **Country** | **Year** | **Slide Sensitivity^1^** | **Treatment arms^2^** | **Age (years)**  **Range** | **Number of**  **patients** | **Reference** |
| --- | --- | --- | --- | --- | --- | --- | --- |
| ADXZX | Gabon | 2005-2006 | 3 | AS-MQ | 1-13 | 71 | ^1^ |
| AJXCU | Cameroon Cote D'Ivoire Senegal | 2008-2009 | 4 | AL, DP | 1-62 | 418 | ^2^ |
| BJUNF | DRC | 2004 | 4 | AS-AQ, AS-SP, SP | 0.5-9 | 180 | ^3^ |
| BKTSC | DRC | 2004 | 3 | AS-AQ, AS-SP | 0.5-4.5 | 174 | ^4^ |
| BMQYT | Mali | 2005 | 4 | AQ-SP, AS-SP, AS-AQcb | 0.5-5 | 400 | ^5^ |
| BQSYZ | Senegal | 2010-2011 | Unknown | AL, AS-MQ | 13-67 | 310 | ^6^ |
| BSCRC | Thailand | 1992-1993 | 3 | AS, AS-MQ, HL, QN | 0.5-49 | 171 | ^7^ |
| BSXFP | Sudan | 2003 | 3 | AS-AQ, AS-SP | 0.5-5 | 161 | ^8^ |
| CCEPC | Uganda | 2002-2004 | 2 | AL | 1-60 | 955 | ^9^ |
| CCXPJ | Cameroon Senegal | 2005 | 4 | AS-AQ | 2-80 | 315 | ^10^ |
| CDCMJ | Myanmar | 2008-2009 | 3 | AL, AS-AQ: FDC,  AS-MQ: FDC, AS-MQ, DP | 1-66 | 808 | ^11^ |
| CFFZN | Thailand | 1993-1994 | 3 | AM-MQ, AS-MQ, MQ | 0-70 | 544 | ^12^ |
| CUDNY | Cambodia | 2002-2003 | 4 | AS-MQ, DP | 1-65 | 454 | ^13^ |
| CXJYT | Kenya Nigeria Tanzania | 2002-2003 | 2 | AL | 0-10 | 310 | ^14^ |
| DADPZ | Uganda | 2007-2008 | 4 | AL, DP | 10-19 | 232 | ^15^ |
| DAXCM | Kenya | 2009 | 1 | AL, DP | 1-10 | 298 | ^16^ |
| DBCXT | Uganda | 2004-2008 | 4 | AL, AQ-SP, AS-AQ | 1-13 | 1453 | ^17^ |
| DFTUS | Senegal | 2011-2012 | Unknown | AL, DP | 10-65 | 240 | Unpublished |
| DPZDY | Thailand | 2008 | 2 | AS-MQ, AS | 19-50 | 40 | ^18^ |
| DYFKY | Afghanistan | 2007-2010 | 4 | AS-SP, DP | 1-70 | 120 | Unpublished |
| EDBXP | Rwanda | 2003-2004 | 2 | AS-SP, AS-AQ, DP | 1-5 | 762 | ^19^ |
| EDPJN | Benin Kenya Mali Mozambique Tanzania | 2006-2007 | 3 | AL | 0-10 | 897 | ^20^ |
| EFTTU | Thailand | 1996-1997 | 2 | AL | 10-62 | 234 | ^21^ |
| EGYMA | Mali | 2005 | 3 | AL, AS-AQ, AS-SP | 0-50 | 778 | ^22^ |
| EKDTF | Mozambique | 2003-2005 | Unknown | AS-SP, SP | 1-65 | 408 | ^23^ |
| EMJMA | Thailand | 1998-2000 | 3 | AS-AV-PG, AS-MQ, AV-PG | 2-70 | 1586 | ^24^ |
| EPDUY | Thailand | 2002-2004 | 4 | AS-DP, AS-MQ, DP | 1-65 | 1027 | ^25^ |
| ESGDS | Thailand | 1993-1995 | 3 | AS, AS-MQ | 0-56 | 85 | ^26^ |
| ETFZC | Uganda | 2003-2004 | 4 | AQ-SP, AS-AQ, CQ-SP | 0.5-47 | 542 | ^27^ |
| EZZJD | Nigeria | 2007-2008 | 2 | AQ-DHA, DHA-SP | 0.5-13 | 99 | Unpublished |
| FAJXQ | India | 2007 | 4 | AQ, AS-AQ:FDC | 0.5-60 | 300 | ^28^ |
| FARTM | Cambodia | 2008-2010 | 3 | AS-MQ | 7-53 | 79 | ^29^ |
| FBDEZ | Guinea-Bissau | 2000-2001 | 4 | AS, AS-CQ, CQ | 0-16 | 471 | ^30^ |
| FEDZY | Liberia | 2008-2009 | 3 | AL, AS-AQ:FDC | 10-70 | 992 | ^31^ |
| FFNAU | Peru | 2003-2005 | 2 | AS-MQ, DP | 5-60 | 522 | ^32^ |
| FHZMF | Gabon Kenya Senegal | 1999-2000 | 2 | AQ, AS-AQ | 0-15 | 935 | ^33^ |
| FMNNB | Thailand | 1995-1996 | 3 | AL, AS-MQ | 2-66 | 606 | ^34^ |
| FNMDP | Sierra Leone | 2004 | 4 | AS-AQ | 6-54 | 126 | ^35^ |
| FXFTC | Uganda | 2004 | 4 | AQ-SP, AS-AQ, CQ-SP | 0.5-9 | 534 | ^27^ |
| GHNKU | Thailand | 1998 | 2 | AL, AS-MQ | 12-71 | 219 | ^36^ |
| GHYRA | Burkina Faso | 2003 | Unknown | CQ, SP | 0.5-5 | 269 | ^37^ |
| GPXJK | Burkina Faso Gabon Mozambique Nigeria Rwanda Uganda Zambia | 2007-2008 | 4 | AL, CDA, AS-AQ:FDC, DP | 0.5-5 | 4112 | ^38^ |
| GQBEK | Thailand | 1993-1996 | 2 | AS, AS-MQ, MQ | 2-16 | 1007 | ^7^ |
| GTEFH | Bangladesh | 2003 | 4 | AL, AS-MQ, CQ-SP | 1-80 | 364 | ^39^ |
| GZQDA | Tanzania | 2007-2008 | 2 | AL | 0-5 | 359 | ^40^ |
| HCEMT | Colombia | 2007-2008 | 1 | AL, AS-MQ, AV-PG | 12-56 | 265 | ^41^ |
| HCGRD | Indonesia | 2011-2012 | 2 | AS-AQ:FDC | 1-59 | 85 | Unpublished |
| HJNDX | Liberia | 2010-2011 | Unknown | AS-AQ:FDC | 0.5-5 | 102 | Unpublished |
| HMPBZ | Sudan | 2009 | Unknown | AL, DP | 1-55 | 71 | ^42^ |
| JGGNM | Burkina Faso Kenya Mozambique Uganda Zambia | 2005 | 2 | AL, DP | 0-7 | 1546 | ^43^ |
| JNCQS | Mali | 2002-2004 | 4 | AS, AS-AQcb, AS-SP | 0.5-56 | 747 | ^44^ |
| JRBRK | Thailand | 2003-2004 | 3 | AS-MQ | 1-68 | 187 | ^45^ |
| JTXEY | Uganda | 2007-2008 | 4 | AL,QN | 0.5-6 | 175 | ^46^ |
| JUTXU | Thailand | 1992 | 3 | AS-MQ, AS, HL, MQ, QN, QN-Tet | 0.5-88 | 321 | ^47^ |
| JYDHH | Uganda | 2002 | 3 | AQ, SP | 0.5-5 | 178 | ^48^ |
| JZCKF | South Africa | 2002 | 2 | AL | 3-66 | 100 | ^49^ |
| KAXED | Gabon | 2003-2004 | 3 | AS-CL, QN-CL | 3-12 | 100 | ^50^ |
| KDDBK | Thailand | 1993-1995 | 3 | AS, AS-MQ | 0-36 | 30 | ^51^ |
| KJGJT | Burkina Faso | 2004-2006 | 2 | AS-AQ:FDC, AS-AQ | 0-6 | 889 | ^52^ |
| KRBXE | Myanmar | 2003-2004 | Unknown | AS-MQ, DP | 1-70 | 652 | ^53^ |
| KTKAB | The Gambia | 2002 | 2 | AL, CQ-SP | 0-10 | 476 | ^54^ |
| KUCMZ | Thailand | 1993-1995 | 3 | AS-MQ, AS | 2-16 | 110 | ^51^ |
| KZBZT | Kenya | 2007 | 3 | AL, DP | 0.5-13 | 146 | ^55^ |
| MCZHT | Uganda | 2006-2007 | 3 | AL, DP | 0.5-9 | 414 | ^56^ |
| MEFSC | Burkina Faso Ghana Kenya Nigeria Tanzania | 2007 | 3 | AL, CDA | 1-14 | 1364 | ^57^ |
| MGEXQ | Colombia | 2000-2004 | 4 | AQ, AS-AQ | 5-62 | 85 | ^58^ |
| MRGRH | Thailand | 2001-2020 | 3 | AS | 0-70 | 2981 | ^59^ |
| MTZZP | Laos | 2004 | 4 | AS-MQ, DP | 1-59 | 220 | ^60^ |
| MZBCX | Sudan | 2003 | 4 | AS-AQ, AS-SP | 0.5-5 | 267 | ^61^ |
| NBSAE | Mali | 2004 | 4 | AL, AS-MQ | 1-70 | 469 | ^62^ |
| NJUCK | Cote D'Ivoire | 2012 | Unknown | AL, AS-AQ | 2-63 | 273 | Unpublished |
| NMMSB | Mozambique | 2003 | Unknown | SP | 1-45 | 25 | ^63^ |
| NRXTM | South Africa | 2004 | 2 | AS-SP | 5-76 | 95 | Unpublished |
| PCGTT | Thailand | 1992 | 3 | HL | 5-50 | 48 | ^64^ |
| PEADD | South Africa | 2002 | 2 | SP | 4-72 | 152 | ^65–67^ |
| PKGFP | Congo | 2004 | 4 | AL, AS-SP, AS-AQ | 0.5-5 | 298 | ^68^ |
| PKSCU | Burkina Faso | 1999-2000 | Unknown | AS-CQ, CQ | 0.5-5 | 300 | ^69^ |
| PNUNE | Uganda | 2005 | 3 | AL | 0-54 | 40 | ^70^ |
| PUEKP | Kenya | 2007 | 3 | AL | 0.5-6 | 267 | ^71^ |
| PXPZK | Mali | 2003-2004 | 4 | AL, AS-SMP | 0.5-63 | 604 | ^72^ |
| QBPQM | Thailand | 2009-2010 | Unknown | AS-MQ | 18-58 | 79 | ^29^ |
| QCKHC | Indonesia | 2005 | 2 | AS-AQ, DP | 1-60 | 219 | ^73^ |
| QFPXT | Uganda | 2002-2003 | 4 | AQ-SP, AS-AQ, CQ-SP | 0.5-65 | 542 | ^27^ |
| QRBRC | DRC | 2008-2009 | 4 | AL, AS-AQ | 0.5-5 | 301 | ^74^ |
| QZJGM | Kenya | 2005 | 4 | AL, DP | 0.5-5 | 469 | ^75^ |
| QZMAG | Indonesia | 2008-2010 | 2 | DP, DP-PQ | 4-66 | 374 | ^76^ |
| RCBGY | Indonesia | 2004-2005 | 2 | AL, DP | 1-60 | 611 | ^77^ |
| RDBXS | Mali | 2009 | 4 | AL | 1-61 | 336 | Unpublished |
| REAJS | Senegal | 2007-2009 | Unknown | AL, AS-AQ:FDC | 0.5-65 | 429 | ^78^ |
| RGDRP | Uganda | 2003-2004 | 4 | AQ-SP, AS-AQ, CQ-SP | 0.5-56 | 541 | ^27^ |
| RHXNJ | Mozambique | 2002 | 2 | SP | 1-64 | 96 | ^63^ |
| RNZFN | Cote d'Ivoire Senegal | 2006-2007 | 4 | AL, AS-AQ | 7-72 | 322 | ^79^ |
| RZENT | Mozambique | 2002 | 2 | SP | 1-58 | 49 | ^63^ |
| SATNJ | Cote D'Ivoire | 2008 | Unknown | AL, AS-AQ:FDC | 0.5-5 | 242 | ^80^ |
| SBCEE | Uganda | 2008-2010 | Unknown | AL, AS-AQ:FDC | 1-65 | 6036 | ^81^ |
| SEFTB | Guinea | 2004 | 4 | AS-AQ, AS-SP | 0.5-5 | 220 | ^82^ |
| SRDFP | Mali | 2010-2011 | 2 | AS | 1-11 | 98 | ^83^ |
| SYFQT | Tanzania | 2004 | 2 | AL, SP | 0.5-10 | 106 | ^84^ |
| SZRDK | Thailand | 2004-2005 | 3 | AS-MQ, AS-MQ:FDC | 0.5-65 | 497 | ^85^ |
| TDFKY | Uganda | 2006 | 4 | AL, DP | 0.5-9 | 421 | ^86^ |
| TETAJ | Laos | 2010 | 3 | AS-AL | 11-66 | 44 | ^87^ |
| TJRQU | Thailand | 1993-1996 | 3 | AM, AS | 0-66 | 440 | ^88^ |
| TYKSC | Cameroon Madagascar Mali Senegal | 2006 | 4 | AL, AS-AQ | 0.5-65 | 940 | ^89^ |
| UBTXH | Liberia | 2008-2009 | 3 | AL, AS-AQ:FDC | 0.5-6 | 299 | ^31^ |
| UEGEJ | Tanzania | 2006 | 1 | AS-SP, AS-SP-PQ | 2-15 | 107 | ^90^ |
| UGPAG | Kenya | 2003-2004 | 1 | AL, AQ-SP, AS-SP, SP | 0.5-11 | 528 | ^91^ |
| UHUBT | Tanzania | 2008 | Unknown | AL | 1-78 | 143 | ^92^ |
| XEDNN | Cameroon  Cote D'Ivoire Senegal | 2007 | 4 | AL, DP | 2-77 | 389 | ^93^ |
| XEKED | Senegal | 2007-2008 | 4 | AL, AS-AQ:FDC | 0.5-8 | 319 | ^94^ |
| XSGGK | Thailand | 1990-1991 | Unknown | HL, MQ, QN | 0-60 | 706 | ^95^ |
| XTGNB | Ethiopia | 2009 | 4 | AL | 0.5-60 | 120 | ^96^ |
| XXFCZ | Tanzania | 2007 | 2 | AL | 0-6 | 244 | ^97^ |
| YGNQS | Senegal | 2010 | 4 | AL, AS-AQ:FDC, DP | 2-63 | 393 | ^98^ |
| YGTAH | Uganda | 2004-2005 | 3 | AL, AS-AQ | 1-9 | 408 | ^99^ |
| YJXJA | Thailand | 1992 | Unknown | HL | 4-24 | 10 | ^95^ |
| YKTFY | Thailand | 1997-1998 | 3 | AL, AS-MQ | 2-63 | 199 | ^64^ |
| YRRSK | Angola | 2004 | 4 | AL, AS-AQ | 7-59 | 137 | ^100^ |
| YYDSM | Benin | 2007 | 4 | AL, AS-AQ:FDC, SP | 0.5-6 | 237 | ^101^ |
| YYMQJ | Ghana | 2002 | 3 | AS-SP, AQ-SP, SP | 0.5-5 | 451 | ^102^ |
| ZQSFC | Colombia | 2001-2005 | Unknown | AL | 17-66 | 45 | ^103^ |
| ZYBXE | Tanzania | 2002-2003 | 4 | AL, AS-AQ | 0-7 | 407 | ^104^ |

**References**

1 Bouyou-Akotet MK, Ramharter M, Ngoungou EB, *et al.* Efficacy and safety of a new pediatric artesunate-mefloquine drug formulation for the treatment of uncomplicated falciparum malaria in Gabon. *Wien Klin Wochenschr* 2010; **122**: 173–8.

2 Menan H, Faye O, Same-Ekobo A, *et al.* Comparative study of the efficacy and tolerability of dihydroartemisinin-piperaquine-trimethoprim versus artemether-lumefantrine in the treatment of uncomplicated Plasmodium falciparum malaria in Cameroon, Ivory Coast and Senegal. *Malar J* 2011; **10**: 185.

3 Bonnet M, Broek I van den, van Herp M, *et al.* Varying efficacy of artesunate+amodiaquine and artesunate+sulphadoxine-pyrimethamine for the treatment of uncomplicated falciparum malaria in the Democratic Republic of Congo: a report of two in-vivo studies. *Malar J* 2009; **8**: 192.

4 Swarthout TD, van den Broek I V, Kayembe G, Montgomery J, Pota H, Roper C. Artesunate + amodiaquine and artesunate + sulphadoxine-pyrimethamine for treatment of uncomplicated malaria in Democratic Republic of Congo: a clinical trial with determination of sulphadoxine and pyrimethamine-resistant haplotypes. *Trop Med Int Health* 2006; **11**: 1503–11.

5 Kayentao K, Maiga H, Newman RD, *et al.* Artemisinin-based combinations versus amodiaquine plus sulphadoxine-pyrimethamine for the treatment of uncomplicated malaria in Faladje, Mali. *Malar J* 2009; **8**: 5.

6 Tine RCK, Faye B, Sylla K, *et al.* Efficacy and tolerability of a new formulation of artesunate-mefloquine for the treatment of uncomplicated malaria in adult in Senegal: open randomized trial. *Malar J* 2012; **11**: 416.

7 Price RN, Nosten F, Luxemburger C, *et al.* Artesunate/mefloquine treatment of multi-drug resistant falciparum malaria. *Trans R Soc Trop Med Hyg* 1997; **91**: 574–7.

8 Hamour S, Melaku Y, Keus K, *et al.* Malaria in the Nuba Mountains of Sudan: baseline genotypic resistance and efficacy of the artesunate plus sulfadoxine-pyrimethamine and artesunate plus amodiaquine combinations. *Trans R Soc Trop Med Hyg* 2005; **99**: 548–54.

9 Piola P, Fogg C, Bajunirwe F, *et al.* Supervised versus unsupervised intake of six-dose artemether-lumefantrine for treatment of acute, uncomplicated Plasmodium falciparum malaria in Mbarara, Uganda: a randomised trial. *Lancet* 2005; **365**: 1467–73.

10 Ndiaye JLA, Faye B, Diouf AM, *et al.* Randomized, comparative study of the efficacy and safety of artesunate plus amodiaquine, administered as a single daily intake versus two daily intakes in the treatment of uncomplicated falciparum malaria. *Malar J* 2008; **7**: 16.

11 Smithuis F, Kyaw MK, Phe O, *et al.* Effectiveness of five artemisinin combination regimens with or without primaquine in uncomplicated falciparum malaria: an open-label randomised trial. *Lancet Infect Dis* 2010; **10**: 673–81.

12 Price RN, Nosten F, Luxemburger C, *et al.* Artesunate versus artemether in combination with mefloquine for the treatment of multidrug-resistant falciparum malaria. *Trans R Soc Trop Med Hyg* 1995; **89**: 523–7.

13 Janssens B, van Herp M, Goubert L, *et al.* A randomized open study to assess the efficacy and tolerability of dihydroartemisinin-piperaquine for the treatment of uncomplicated falciparum malaria in Cambodia. *Trop Med Int Health* 2007; **12**: 251–9.

14 Falade C, Makanga M, Premji Z, Ortmann C-E, Stockmeyer M, de Palacios PI. Efficacy and safety of artemether-lumefantrine (Coartem) tablets (six-dose regimen) in African infants and children with acute, uncomplicated falciparum malaria. *Trans R Soc Trop Med Hyg* 2005; **99**: 459–67.

15 Arinaitwe E, Sandison TG, Wanzira H, *et al.* Artemether-lumefantrine versus dihydroartemisinin-piperaquine for falciparum malaria: a longitudinal, randomized trial in young Ugandan children. *Clin Infect Dis* 2009; **49**: 1629–37.

16 Sawa P, Shekalaghe S a, Drakeley CJ, *et al.* Malaria transmission after artemether-lumefantrine and dihydroartemisinin-piperaquine: a randomized trial. *J Infect Dis* 2013; **207**: 1637–45.

17 Dorsey G, Staedke S, Clark TD, *et al.* Combination therapy for uncomplicated falciparum malaria in Ugandan children: a randomized trial. *JAMA* 2007; **297**: 2210–9.

18 Dondorp AM, Nosten F, Yi P, *et al.* Artemisinin resistance in Plasmodium falciparum malaria. *N Engl J Med* 2009; **361**: 455–67.

19 Karema C, Fanello CI, van Overmeir C, *et al.* Safety and efficacy of dihydroartemisinin/piperaquine (Artekin) for the treatment of uncomplicated Plasmodium falciparum malaria in Rwandan children. *Trans R Soc Trop Med Hyg* 2006; **100**: 1105–11.

20 Abdulla S, Sagara I, Borrmann S, *et al.* Efficacy and safety of artemether-lumefantrine dispersible tablets compared with crushed commercial tablets in African infants and children with uncomplicated malaria: a randomised, single-blind, multicentre trial. *Lancet* 2008; **372**: 1819–27.

21 Van Vugt M, Wilairatana P, Gemperli B, *et al.* Efficacy of six doses of artemether-lumefantrine (benflumetol) in multidrug-resistant Plasmodium falciparum malaria. *Am J Trop Med Hyg* 1999; **60**: 936–42.

22 Sagara I, Fofana B, Gaudart J, *et al.* Repeated artemisinin-based combination therapies in a malaria hyperendemic area of Mali: efficacy, safety, and public health impact. *Am J Trop Med Hyg* 2012; **87**: 50–6.

23 Allen EN, Little F, Camba T, *et al.* Efficacy of sulphadoxine-pyrimethamine with or without artesunate for the treatment of uncomplicated Plasmodium falciparum malaria in southern Mozambique: a randomized controlled trial. *Malar J* 2009; **8**: 141.

24 van Vugt M, Leonardi E, Phaipun L, *et al.* Treatment of uncomplicated multidrug-resistant falciparum malaria with artesunate-atovaquone-proguanil. *Clin Infect Dis* 2002; **35**: 1498–504.

25 Ashley EA, Krudsood S, Phaiphun L, *et al.* Randomized, controlled dose-optimization studies of dihydroartemisinin-piperaquine for the treatment of uncomplicated multidrug-resistant falciparum malaria in Thailand. *J Infect Dis* 2004; **190**: 1773–82.

26 Price RN, Uhlemann A-C, van Vugt M, *et al.* Molecular and pharmacological determinants of the therapeutic response to artemether-lumefantrine in multidrug-resistant Plasmodium falciparum malaria. *Clin Infect Dis* 2006; **42**: 1570–7.

27 Yeka A, Banek K, Bakyaita N, *et al.* Artemisinin versus nonartemisinin combination therapy for uncomplicated malaria: randomized clinical trials from four sites in Uganda. *PLoS Med* 2005; **2**: e190.

28 Anvikar AR, Sharma B, Shahi BH, *et al.* Artesunate-amodiaquine fixed dose combination for the treatment of Plasmodium falciparum malaria in India. *Malar J* 2012; **11**: 97.

29 Das D, Tripura R, Phyo AP, *et al.* Effect of high-dose or split-dose artesunate on parasite clearance in artemisinin-resistant falciparum malaria. *Clin Infect Dis* 2013; **56**: e48–58.

30 Kofoed P-E, Poulsen A, Có F, Hedegaard K, Aaby P, Rombo L. No benefits from combining chloroquine with artesunate for three days for treatment of Plasmodium falciparum in Guinea-Bissau. *Trans R Soc Trop Med Hyg* 2003; **97**: 429–33.

31 Schramm B, Valeh P, Baudin E, *et al.* Efficacy of artesunate-amodiaquine and artemether-lumefantrine fixed-dose combinations for the treatment of uncomplicated Plasmodium falciparum malaria among children aged six to 59 months in Nimba County, Liberia: an open-label randomized non-inferiority. *Malar J* 2013; **12**: 251.

32 Grande T, Bernasconi A, Erhart A, *et al.* A randomised controlled trial to assess the efficacy of dihydroartemisinin-piperaquine for the treatment of uncomplicated falciparum malaria in Peru. *PLoS One* 2007; **2**: e1101.

33 Adjuik M, Agnamey P, Babiker A, *et al.* Amodiaquine-artesunate versus amodiaquine for uncomplicated Plasmodium falciparum malaria in African children: a randomised, multicentre trial. *Lancet* 2002; **359**: 1365–72.

34 van Vugt M, Brockman A, Gemperli B, *et al.* Randomized comparison of artemether-benflumetol and artesunate-mefloquine in treatment of multidrug-resistant falciparum malaria. *Antimicrob Agents Chemother* 1998; **42**: 135–9.

35 Grandesso F, Hagerman A, Kamara S, *et al.* Low efficacy of the combination artesunate plus amodiaquine for uncomplicated falciparum malaria among children under 5 years in Kailahun, Sierra Leone. *Trop Med Int Health* 2006; **11**: 1017–21.

36 Lefèvre G, Looareesuwan S, Treeprasertsuk S, *et al.* A clinical and pharmacokinetic trial of six doses of artemether-lumefantrine for multidrug-resistant Plasmodium falciparum malaria in Thailand. *Am J Trop Med Hyg* 2001; **64**: 247–56.

37 Gansané A, Nébié I, Soulama I, *et al.* [Change of antimalarial first-line treatment in Burkina Faso in 2005]. *Bull Soc Pathol Exot* 2009; **102**: 31–5.

38 A head-to-head comparison of four artemisinin-based combinations for treating uncomplicated malaria in African children: a randomized trial. *PLoS Med* 2011; **8**: e1001119.

39 van den Broek I V, Maung UA, Peters A, *et al.* Efficacy of chloroquine + sulfadoxine--pyrimethamine, mefloquine + artesunate and artemether + lumefantrine combination therapies to treat Plasmodium falciparum malaria in the Chittagong Hill Tracts, Bangladesh. *Trans R Soc Trop Med Hyg* 2005; **99**: 727–35.

40 Ngasala BE, Malmberg M, Carlsson AM, *et al.* Efficacy and effectiveness of artemether-lumefantrine after initial and repeated treatment in children <5 years of age with acute uncomplicated Plasmodium falciparum malaria in rural Tanzania: a randomized trial. *Clin Infect Dis* 2011; **52**: 873–82.

41 Carrasquilla G, Barón C, Monsell EM, *et al.* Randomized, prospective, three-arm study to confirm the auditory safety and efficacy of artemether-lumefantrine in Colombian patients with uncomplicated Plasmodium falciparum malaria. *Am J Trop Med Hyg* 2012; **86**: 75–83.

42 Adam I, Salah MT, Eltahir HG, Elhassan AH, Elmardi KA, Malik EM. Dihydroartemisinin-piperaquine versus artemether-lumefantrine, in the treatment of uncomplicated Plasmodium falciparum malaria in central Sudan. *Ann Trop Med Parasitol* 2010; **104**: 319–26.

43 Bassat Q, Mulenga M, Tinto H, *et al.* Dihydroartemisinin-piperaquine and artemether-lumefantrine for treating uncomplicated malaria in African children: a randomised, non-inferiority trial. *PLoS One* 2009; **4**: e7871.

44 Djimdé AA, Fofana B, Sagara I, *et al.* Efficacy, safety, and selection of molecular markers of drug resistance by two ACTs in Mali. *Am J Trop Med Hyg* 2008; **78**: 455–61.

45 Carrara VI, Zwang J, Ashley EA, *et al.* Changes in the treatment responses to artesunate-mefloquine on the northwestern border of Thailand during 13 years of continuous deployment. *PLoS One* 2009; **4**: e4551.

46 Achan J, Tibenderana JK, Kyabayinze D, *et al.* Effectiveness of quinine versus artemether-lumefantrine for treating uncomplicated falciparum malaria in Ugandan children: randomised trial. *BMJ* 2009; **339**: b2763.

47 Nosten F, Luxemburger C, ter Kuile FO, *et al.* Treatment of multidrug-resistant Plasmodium falciparum malaria with 3-day artesunate-mefloquine combination. *J Infect Dis* 1994; **170**: 971–7.

48 Checchi F, Piola P, Kosack C, *et al.* Antimalarial efficacy of sulfadoxine-pyrimethamine, amodiaquine and a combination of chloroquine plus sulfadoxine-pyrimethamine in Bundi Bugyo, western Uganda. *Trop Med Int Health* 2004; **9**: 445–50.

49 Barnes KI, Durrheim DN, Little F, *et al.* Effect of artemether-lumefantrine policy and improved vector control on malaria burden in KwaZulu-Natal, South Africa. *PLoS Med* 2005; **2**: e330.

50 Ramharter M, Oyakhirome S, Klein Klouwenberg P, *et al.* Artesunate-clindamycin versus quinine-clindamycin in the treatment of Plasmodium falciparum malaria: a randomized controlled trial. *Clin Infect Dis* 2005; **40**: 1777–84.

51 Price R, Luxemburger C, van Vugt M, *et al.* Artesunate and mefloquine in the treatment of uncomplicated multidrug-resistant hyperparasitaemic falciparum malaria. *Trans R Soc Trop Med Hyg* 1998; **92**: 207–11.

52 Sirima SB, Tiono AB, Gansané A, *et al.* The efficacy and safety of a new fixed-dose combination of amodiaquine and artesunate in young African children with acute uncomplicated Plasmodium falciparum. *Malar J* 2009; **8**: 48.

53 Smithuis F, Kyaw MK, Phe O, *et al.* Efficacy and effectiveness of dihydroartemisinin-piperaquine versus artesunate-mefloquine in falciparum malaria: an open-label randomised comparison. *Lancet* 2006; **367**: 2075–85.

54 Sutherland CJ, Ord R, Dunyo S, *et al.* Reduction of malaria transmission to Anopheles mosquitoes with a six-dose regimen of co-artemether. *PLoS Med* 2005; **2**: e92.

55 Mens PF, Sawa P, van Amsterdam SM, *et al.* A randomized trial to monitor the efficacy and effectiveness by QT-NASBA of artemether-lumefantrine versus dihydroartemisinin-piperaquine for treatment and transmission control of uncomplicated Plasmodium falciparum malaria in western Kenya. *Malar J* 2008; **7**: 237.

56 Yeka A, Dorsey G, Kamya MR, *et al.* Artemether-lumefantrine versus dihydroartemisinin-piperaquine for treating uncomplicated malaria: a randomized trial to guide policy in Uganda. *PLoS One* 2008; **3**: e2390.

57 Premji Z, Umeh RE, Owusu-Agyei S, *et al.* Chlorproguanil-dapsone-artesunate versus artemether-lumefantrine: a randomized, double-blind phase III trial in African children and adolescents with uncomplicated Plasmodium falciparum malaria. *PLoS One* 2009; **4**: e6682.

58 Osorio L, Gonzalez I, Olliaro P, Taylor WRJ. Artemisinin-based combination therapy for uncomplicated Plasmodium falciparum malaria in Colombia. *Malar J* 2007; **6**: 25.

59 Phyo AP, Nkhoma S, Stepniewska K, *et al.* Emergence of artemisinin-resistant malaria on the western border of Thailand: a longitudinal study. *Lancet* 2012; **379**: 1960–6.

60 Mayxay M, Thongpraseuth V, Khanthavong M, *et al.* An open, randomized comparison of artesunate plus mefloquine vs. dihydroartemisinin-piperaquine for the treatment of uncomplicated Plasmodium falciparum malaria in the Lao People’s Democratic Republic (Laos). *Trop Med Int Health* 2006; **11**: 1157–65.

61 van den Broek I, Amsalu R, Balasegaram M, *et al.* Efficacy of two artemisinin combination therapies for uncomplicated falciparum malaria in children under 5 years, Malakal, Upper Nile, Sudan. *Malar J* 2005; **4**: 14.

62 Sagara I, Diallo A, Kone M, *et al.* A randomized trial of artesunate-mefloquine versus artemether-lumefantrine for treatment of uncomplicated Plasmodium falciparum malaria in Mali. *Am J Trop Med Hyg* 2008; **79**: 655–61.

63 Barnes KI, Little F, Smith PJ, Evans A, Watkins WM, White NJ. Sulfadoxine-pyrimethamine pharmacokinetics in malaria: pediatric dosing implications. *Clin Pharmacol Ther* 2006; **80**: 582–96.

64 van Vugt M, Looareesuwan S, Wilairatana P, *et al.* Artemether-lumefantrine for the treatment of multidrug-resistant falciparum malaria. *Trans R Soc Trop Med Hyg* 2000; **94**: 545–8.

65 Mabuza A, Govere J, La Grange K, *et al.* Therapeutic efficacy of sulfadoxine-pyrimethamine for Plasmodium falciparum malaria. *S Afr Med J* 2005; **95**: 346–9.

66 Barnes KI, Little F, Smith PJ, Evans A, Watkins WM, White NJ. Sulfadoxine-pyrimethamine pharmacokinetics in malaria: pediatric dosing implications. *Clin Pharmacol Ther* 2006; **80**: 582–96.

67 Barnes KI, Little F, Mabuza A, *et al.* Increased gametocytemia after treatment: an early parasitological indicator of emerging sulfadoxine-pyrimethamine resistance in falciparum malaria. *J Infect Dis* 2008; **197**: 1605–13.

68 van den Broek I, Kitz C, Al Attas S, Libama F, Balasegaram M, Guthmann J-P. Efficacy of three artemisinin combination therapies for the treatment of uncomplicated Plasmodium falciparum malaria in the Republic of Congo. *Malar J* 2006; **5**: 113.

69 Sirima SB, Tiono AB, Konaté A, *et al.* Efficacy of artesunate plus chloroquine for the treatment of uncomplicated malaria in children in Burkina Faso: a double-blind, randomized, controlled trial. *Trans R Soc Trop Med Hyg* 2003; **97**: 345–9.

70 Fogg C, Twesigye R, Batwala V, *et al.* Assessment of three new parasite lactate dehydrogenase (pan-pLDH) tests for diagnosis of uncomplicated malaria. *Trans R Soc Trop Med Hyg* 2008; **102**: 25–31.

71 Juma EA, Obonyo CO, Akhwale WS, Ogutu BR. A randomized, open-label, comparative efficacy trial of artemether-lumefantrine suspension versus artemether-lumefantrine tablets for treatment of uncomplicated Plasmodium falciparum malaria in children in western Kenya. *Malar J* 2008; **7**: 262.

72 Sagara I, Dicko A, Djimde A, *et al.* A randomized trial of artesunate-sulfamethoxypyrazine-pyrimethamine versus artemether-lumefantrine for the treatment of uncomplicated Plasmodium falciparum malaria in Mali. *Am J Trop Med Hyg* 2006; **75**: 630–6.

73 Hasugian AR, Purba HLE, Kenangalem E, *et al.* Dihydroartemisinin-piperaquine versus artesunate-amodiaquine: superior efficacy and posttreatment prophylaxis against multidrug-resistant Plasmodium falciparum and Plasmodium vivax malaria. *Clin Infect Dis* 2007; **44**: 1067–74.

74 Espié E, Lima A, Atua B, *et al.* Efficacy of fixed-dose combination artesunate-amodiaquine versus artemether-lumefantrine for uncomplicated childhood Plasmodium falciparum malaria in Democratic Republic of Congo: a randomized non-inferiority trial. *Malar J* 2012; **11**: 174.

75 Borrmann S, Sasi P, Mwai L, *et al.* Declining responsiveness of Plasmodium falciparum infections to artemisinin-based combination treatments on the Kenyan coast. *PLoS One* 2011; **6**: e26005.

76 Sutanto I, Suprijanto S, Kosasih A, *et al.* The effect of primaquine on gametocyte development and clearance in the treatment of uncomplicated falciparum malaria with dihydroartemisinin-piperaquine in South sumatra, Western indonesia: an open-label, randomized, controlled trial. *Clin Infect Dis* 2013; **56**: 685–93.

77 Ratcliff A, Siswantoro H, Kenangalem E, *et al.* Two fixed-dose artemisinin combinations for drug-resistant falciparum and vivax malaria in Papua, Indonesia: an open-label randomised comparison. *Lancet* 2007; **369**: 757–65.

78 Ndiaye J-LA, Faye B, Gueye A, *et al.* Repeated treatment of recurrent uncomplicated Plasmodium falciparum malaria in Senegal with fixed-dose artesunate plus amodiaquine versus fixed-dose artemether plus lumefantrine: a randomized, open-label trial. *Malar J* 2011; **10**: 237.

79 Faye B, Offianan AT, Ndiaye JL, *et al.* Efficacy and tolerability of artesunate-amodiaquine (Camoquin plus) versus artemether-lumefantrine (Coartem) against uncomplicated Plasmodium falciparum malaria: multisite trial in Senegal and Ivory Coast. *Trop Med Int Health* 2010; **15**: 608–13.

80 Offianan AT, Assi SB, Coulibaly A, N’guessan LT, Ako AA, Kadjo FK, San MK PL. Assessment of the efficacy of first-line antimalarial drugs after 5 years of deployment by the National Malaria Control Programme in Côte d’Ivoire. *Volume* 2011; **2011:3**: Pages 67—76.

81 Yeka A, Lameyre V, Afizi K, *et al.* Efficacy and safety of fixed-dose artesunate-amodiaquine vs. artemether-lumefantrine for repeated treatment of uncomplicated malaria in Ugandan children. *PLoS One* 2014; **9**: e113311.

82 Bonnet M, Roper C, Félix M, Coulibaly L, Kankolongo GM, Guthmann JP. Efficacy of antimalarial treatment in Guinea: in vivo study of two artemisinin combination therapies in Dabola and molecular markers of resistance to sulphadoxine-pyrimethamine in N’Zérékoré. *Malar J* 2007; **6**: 54.

83 Maiga AW, Fofana B, Sagara I, *et al.* No evidence of delayed parasite clearance after oral artesunate treatment of uncomplicated falciparum malaria in Mali. *Am J Trop Med Hyg* 2012; **87**: 23–8.

84 Martensson A, Ngasala B, Ursing J, *et al.* Influence of consecutive-day blood sampling on polymerase chain reaction-adjusted parasitological cure rates in an antimalarial-drug trial conducted in Tanzania. *J Infect Dis* 2007; **195**: 597–601.

85 Ashley EA, Lwin KM, McGready R, *et al.* An open label randomized comparison of mefloquine-artesunate as separate tablets vs. a new co-formulated combination for the treatment of uncomplicated multidrug-resistant falciparum malaria in Thailand. *Trop Med Int Health* 2006; **11**: 1653–60.

86 Kamya MR, Yeka A, Bukirwa H, *et al.* Artemether-lumefantrine versus dihydroartemisinin-piperaquine for treatment of malaria: a randomized trial. *PLoS Clin Trials* 2007; **2**: e20.

87 Mayxay M, Khanthavong M, Chanthongthip O, *et al.* No evidence for spread of Plasmodium falciparum artemisinin resistance to Savannakhet Province, Southern Laos. *Am J Trop Med Hyg* 2012; **86**: 403–8.

88 Price R, van Vugt M, Nosten F, *et al.* Artesunate versus artemether for the treatment of recrudescent multidrug-resistant falciparum malaria. *Am J Trop Med Hyg* 1998; **59**: 883–8.

89 Ndiaye JL, Randrianarivelojosia M, Sagara I, *et al.* Randomized, multicentre assessment of the efficacy and safety of ASAQ--a fixed-dose artesunate-amodiaquine combination therapy in the treatment of uncomplicated Plasmodium falciparum malaria. *Malar J* 2009; **8**: 125.

90 Shekalaghe S, Drakeley C, Gosling R, *et al.* Primaquine clears submicroscopic Plasmodium falciparum gametocytes that persist after treatment with sulphadoxine-pyrimethamine and artesunate. *PLoS One* 2007; **2**: e1023.

91 Bousema JT, Schneider P, Gouagna LC, *et al.* Moderate effect of artemisinin-based combination therapy on transmission of Plasmodium falciparum. *J Infect Dis* 2006; **193**: 1151–9.

92 Hodel EM, Kabanywanyi AM, Malila A, *et al.* Residual antimalarials in malaria patients from Tanzania--implications on drug efficacy assessment and spread of parasite resistance. *PLoS One* 2009; **4**: e8184.

93 Yavo W, Faye B, Kuete T, *et al.* Multicentric assessment of the efficacy and tolerability of dihydroartemisinin-piperaquine compared to artemether-lumefantrine in the treatment of uncomplicated Plasmodium falciparum malaria in sub-Saharan Africa. *Malar J* 2011; **10**: 198.

94 Faye B, Ndiaye JL, Tine R, *et al.* A randomized trial of artesunate mefloquine versus artemether lumefantrine for the treatment of uncomplicated Plasmodium falciparum malaria in Senegalese children. *Am J Trop Med Hyg* 2010; **82**: 140–4.

95 ter Kuile FO, Dolan G, Nosten F, *et al.* Halofantrine versus mefloquine in treatment of multidrug-resistant falciparum malaria. *Lancet* 1993; **341**: 1044–9.

96 Hwang J, Alemayehu BH, Hoos D, *et al.* In vivo efficacy of artemether-lumefantrine against uncomplicated Plasmodium falciparum malaria in Central Ethiopia. *Malar J* 2011; **10**: 209.

97 Ngasala BE, Malmberg M, Carlsson AM, *et al.* Effectiveness of artemether-lumefantrine provided by community health workers in under-five children with uncomplicated malaria in rural Tanzania: an open label prospective study. *Malar J* 2011; **10**: 64.

98 Sylla K, Abiola A, Tine RCK, *et al.* Monitoring the efficacy and safety of three artemisinin based-combinations therapies in Senegal: results from two years surveillance. *BMC Infect Dis* 2013; **13**: 598.

99 Bukirwa H, Yeka A, Kamya MR, *et al.* Artemisinin combination therapies for treatment of uncomplicated malaria in Uganda. *PLoS Clin Trials* 2006; **1**: e7.

100 Guthmann J-P, Cohuet S, Rigutto C, *et al.* High efficacy of two artemisinin-based combinations (artesunate + amodiaquine and artemether + lumefantrine) in Caala, Central Angola. *Am J Trop Med Hyg* 2006; **75**: 143–5.

101 Faucher J-F, Aubouy A, Adeothy A, *et al.* Comparison of sulfadoxine-pyrimethamine, unsupervised artemether-lumefantrine, and unsupervised artesunate-amodiaquine fixed-dose formulation for uncomplicated plasmodium falciparum malaria in Benin: a randomized effectiveness noninferiority trial. *J Infect Dis* 2009; **200**: 57–65.

102 Mockenhaupt FP, Teun Bousema J, Eggelte TA, *et al.* Plasmodium falciparum dhfr but not dhps mutations associated with sulphadoxine-pyrimethamine treatment failure and gametocyte carriage in northern Ghana. *Trop Med Int Health* 2005; **10**: 901–8.

103 Hatz C, Soto J, Nothdurft HD, *et al.* Treatment of acute uncomplicated falciparum malaria with artemether-lumefantrine in nonimmune populations: a safety, efficacy, and pharmacokinetic study. *Am J Trop Med Hyg* 2008; **78**: 241–7.

104 Mårtensson A, Strömberg J, Sisowath C, *et al.* Efficacy of artesunate plus amodiaquine versus that of artemether-lumefantrine for the treatment of uncomplicated childhood Plasmodium falciparum malaria in Zanzibar, Tanzania. *Clin Infect Dis* 2005; **41**: 1079–86.
