# Supplementary figures and images for: Gametocyte carriage in uncomplicated Plasmodium falciparum malaria following treatment with artemisinin combination therapy: a systematic review and meta-analysis of individual patient data
Source: BMC Med. 2016 May 24;14:79. doi: 10.1186/s12916-016-0621-7 (PMC4879753; doi:10.1186/s12916-016-0621-7)

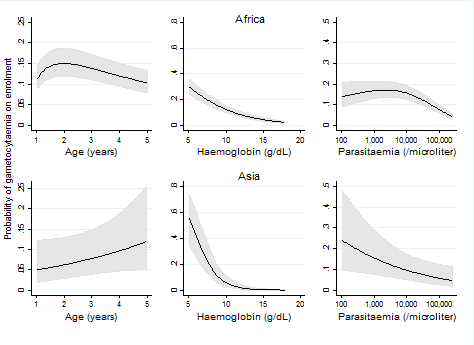

Supplement: Additional file 2: Figure S1. — Relationship between gametocytaemia on enrolment and baseline haemoglobin concentration, parasitaemia and patient age. The predicted probability of gametocyte carriage at enrolment is plotted from the multivariate model; the line indicates the best fit, the shaded area the 95 % CI. Only patients from studies with gametocyte detection sensitivity in category 1 or 2 were used for this analysis (≥ 100 high power fields or ≥ 1000 WBC examined specifically for gametocytes). For the analysis on age, only children < 5 years of age were included in the analysis. (TIF 480 kb) [file 12916_2016_621_MOESM2_ESM.tif]

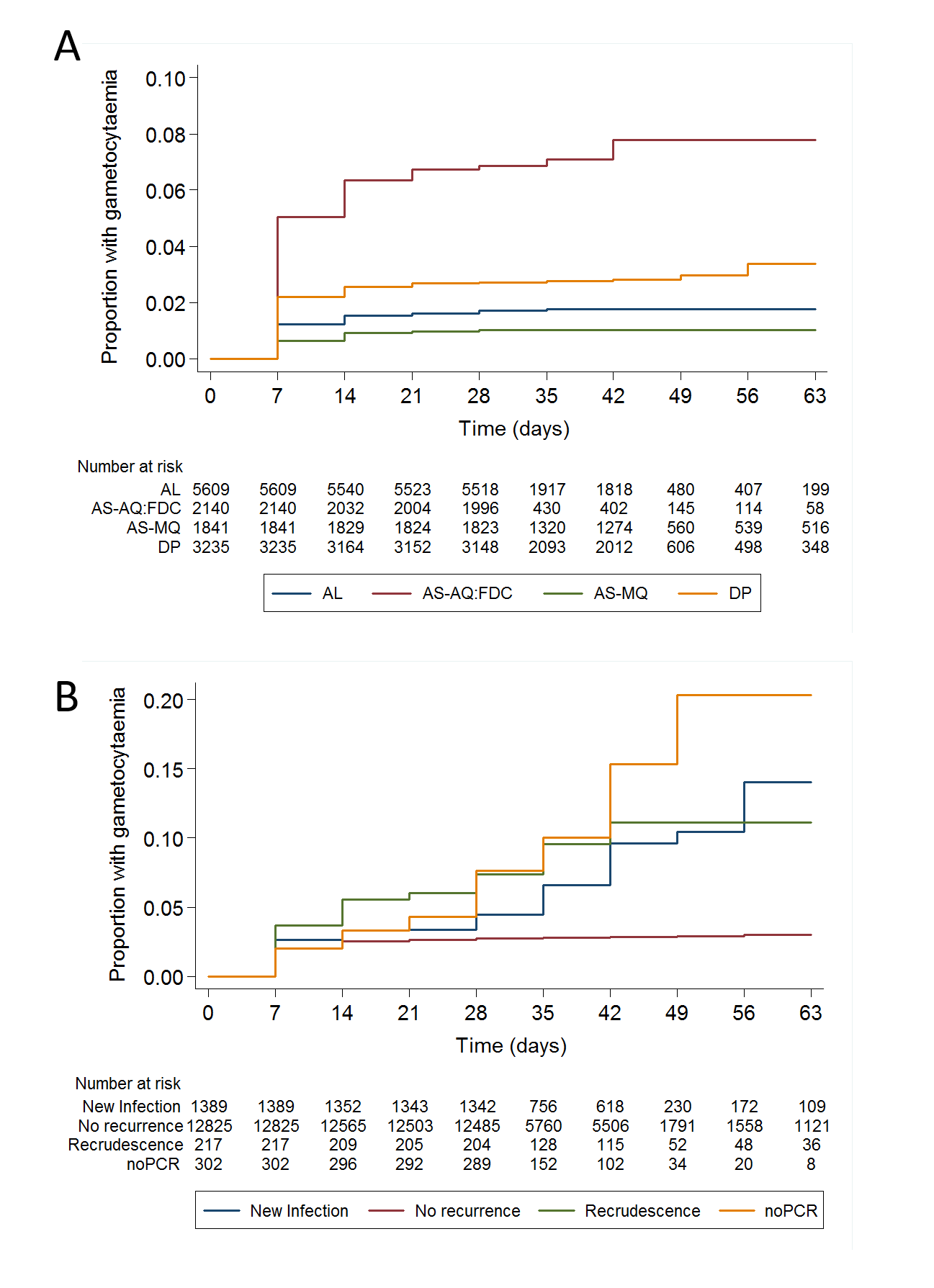

Supplement: Additional file 9: Figure S2. — Development of gametocytaemia after treatment evaluated in patients with no gametocytaemia on enrolment and full 28-day follow-up. A: Development of gametocytaemia by artemisinin combination therapy. B: Development of gametocytaemia by treatment outcome. (TIF 284 kb) [file 12916_2016_621_MOESM9_ESM.tif]
