# Supplementary material for: Gametocyte carriage in uncomplicated Plasmodium falciparum malaria following treatment with artemisinin combination therapy: a systematic review and meta-analysis of individual patient data
Source: BMC Med. 2016 May 24;14:79. doi: 10.1186/s12916-016-0621-7 (PMC4879753; doi:10.1186/s12916-016-0621-7)
Supplement: Additional file 3: Table S2. — Independent risk factors for the prevalence of gametocytaemia at enrolment in children aged 1–5 years. Logistic multivariable analysis by region with prevalence of gametocytaemia at enrolment as dependent variable. Nobs, Number of observations; Npos, Number of positive observations. The relationship between gametocyte prevalence at enrolment and age is statistically significant (P < 0.001) although not linear, see Additional file 2: Figure S1. Malnutrition (underweight) was not an independent predictor (AOR, 1.11; 95 % CI, 0.99–1.26; P = 0.083) in Africa and (AOR, 0.92; 95 % CI, 0.47–0.83; P = 0.823) in Asia, after adjustment for age, haemoglobin, parasitaemia and fever (after polynomial transformations, as presented in Additional file 2: Figure S1). (DOC 28 kb) [file 12916_2016_621_MOESM3_ESM.doc]

**Supplementary Table S2. Independent risk factors for prevalence of gametocytaemia at enrolment in children 1- 5 years of age.**

|  | Africa | | | Asia | | |
| --- | --- | --- | --- | --- | --- | --- |
| Parameter | Nobs/Npos (%) | AOR (95% CI) | P-value | Nobs/Npos (%) | AOR (95% CI) | P-value |
| **Multivariable model** | 16735/2505 (15.0) |  |  | 765/100 (13.1) |  |  |
| Age (years) |  | 0.973 (0.928 – 1.020) | 0.255 |  | 1.298 (0.977-1.726) | 0.072 |
| Derived Haemoglobin |  | 0.805 (0.781 – 0.829) | <0.001 |  | 0.553 (0.479 – 0.640) | <0.001 |
| Log_10_ Parasitaemia |  | 0.585 (0.536 – 0.637) | <0.001 |  | 0.747 (0.539-1.034) | 0.078 |
| Fever |  | 0.637 (0.575 – 0.706) | <0.001 |  | 0.609 (0.358 – 1.037) | 0.068 |
