# Supplementary material for: Gametocyte carriage in uncomplicated Plasmodium falciparum malaria following treatment with artemisinin combination therapy: a systematic review and meta-analysis of individual patient data
Source: BMC Med. 2016 May 24;14:79. doi: 10.1186/s12916-016-0621-7 (PMC4879753; doi:10.1186/s12916-016-0621-7)
Supplement: Additional file 4: Table S3. — The effect of treatment dosing on the appearance of gametocytaemia in participants without microscopically detected gametocytaemia before treatment (time to gametocytaemia) and clearance of gametocytaemia in participants with gametocytaemia at enrolment (time to clearance). All analyses of time to clearance are adjusted for log of the initial gametocyte density. Nobs, Number of observations; Npos, Number of positive observations. Under-dosed defined as ≤ 8.4 mg/kg artemether dose in AL, < 6 mg/kg dose of artesunate or DHA in other regimens [19]. In the multivariate model estimates are adjusted for other covariates, for time to gametocytaemia: covariates identified in the full final model presented in Table 4; for time to clearance: ACT, since no other covariates other than ACT were identified in the final model there were no multivariate models fitted within each ACT. ND, No data, HR could not be estimated as there were no patients with gametocytaemia in the under-dose/low-dose group. (DOC 86 kb) [file 12916_2016_621_MOESM4_ESM.doc]

**Supplementary Table S3. The effect of treatment dosing on appearance of gametocytaemia in participants without microscopically detected gametocytaemia before treatment (time to gametocytaemia) and gametocytaemia clearance in participants with gametocytaemia at enrolment (time to gametocytaemia clearance).**

|  | Time to gametocytaemia | | | | | Time to gametocytaemia clearance | | | | |
| --- | --- | --- | --- | --- | --- | --- | --- | --- | --- | --- |
| Parameter | Nobs | Npos | per | HR (95%CI) | p-value | Nobs | Npos | per | HR (95%CI) | p-value |
| **Univariable analysis** |  |  |  |  |  |  |  |  |  |  |
| Artemisinin Dose  All | 17986 | 358 | 2.0 | 0.996(0.963 - 1.031) | 0.838 | 1567 | 1285 | 82.0 | 1.031 (1.011 - 1.050) | 0.002 |
| AL | 8432 | 97 | 1.2 | 0.972 (0.893 - 1.058) | 0.518 | 491 | 413 | 84.1 | 1.022 (0.985 - 1.060) | 0.242 |
| AS-AQ: FDC | 2915 | 151 | 5.2 | 1.063 (1.016 - 1.112) | 0.008 | 263 | 223 | 84.8 | 0.993 (0.944 - 1.045) | 0.787 |
| AS-MQ | 2859 | 17 | 0.6 | 1.049 (0.948 - 1.160) | 0.353 | 251 | 203 | 80.9 | 0.984 (0.914 - 1.059) | 0.667 |
| DP | 3780 | 93 | 2.5 | 0.884 (0.757 - 1.033) | 0.120 | 562 | 446 | 79.4 | 0.987 (0.924 - 1.054) | 0.693 |
| Partner Drug Dose  AL | 8432 | 97 | 1.2 | 0.995 (0.981 - 1.010) | 0.518 | 491 | 413 | 84.1 | 1.004 (0.998 - 1.010) | 0.242 |
| AS-AQ:FDC | 2915 | 151 | 5.2 | 1.023 (1.006 - 1.040) | 0.008 | 263 | 223 | 84.8 | 0.997 (0.979 - 1.016) | 0.787 |
| AS-MQ | 2682 | 17 | 0.6 | 0.993 (0.889 - 1.109) | 0.894 | 250 | 202 | 80.8 | 1.002 (0.971 - 1.035) | 0.885 |
| DP | 3780 | 93 | 2.5 | 0.985 (0.966 - 1.004) | 0.120 | 562 | 446 | 79.4 | 0.998 (0.990 - 1.007) | 0.693 |
| Underdose  All | 17986 | 358 | 2.0 | 1.117 (0.734 - 1.700) | 0.606 | 1567 | 1285 | 82.0 | 0.831 (0.675 - 1.024) | 0.082 |
| AL | 8432 | 97 | 1.2 | 0.698 (0.262 - 1.863) | 0.473 | 491 | 413 | 84.1 | 0.883 (0.599 - 1.302) | 0.531 |
| AS-AQ:FDC | 2915 | 151 | 5.2 | ND |  | 263 | 223 | 84.8 | ND |  |
| AS-MQ | 2859 | 17 | 0.6 | ND |  | 251 | 203 | 80.9 | 1.603 (0.182 - 14.104) | 0.671 |
| DP | 3780 | 93 | 2.5 | 1.255 (0.751 - 2.097) | 0.386 | 562 | 446 | 79.4 | 0.889 (0.684 - 1.155) | 0.377 |
| Low Dose (< 8mg/kg)  All | 17986 | 358 | 2.0 | 1.638 (1.202 - 2.232) | 0.002 | 1567 | 1285 | 82.0 | 0.847 (0.735 - 0.976) | 0.022 |
| AL | 8432 | 97 | 1.2 | 0.409 (0.091 - 1.843) | 0.244 | 491 | 413 | 84.1 | 0.804 (0.493 - 1.311) | 0.382 |
| AS-AQ:FDC | 2915 | 151 | 5.2 | 1.325 (0.326 - 5.390) | 0.694 | 263 | 223 | 84.8 | 1.081 (0.258 - 4.526) | 0.915 |
| AS-MQ | 2859 | 17 | 0.6 | ND |  | 251 | 203 | 80.9 | 1.259 (0.427 - 3.716) | 0.677 |
| DP | 3780 | 93 | 2.5 | 1.569 (0.933 - 2.638) | 0.090 | 562 | 446 | 79.4 | 1.095 (0.883 - 1.357) | 0.411 |
| **Multivariable analysis** |  |  |  |  |  |  |  |  |  |  |
| Artemisinin Dose  ALL | 13374 | 284 | 2.1 | 0.993 (0.952 - 1.036) | 0.733 |  |  |  |  |  |
| AL | 6088 | 80 | 1.3 | 0.947 (0.855 - 1.049) | 0.301 |  |  |  |  |  |
| AS-AQ: FDC | 1671 | 132 | 7.9 | 1.026 (0.975 - 1.080) | 0.322 |  |  |  |  |  |
| AS-MQ | 2373 | 11 | 0.5 | 1.048 (0.931 - 1.179) | 0.439 |  |  |  |  |  |
| DP | 3242 | 61 | 1.9 | 0.787 (0.635 - 0.976) | 0.029 |  |  |  |  |  |
| Partner Drug Dose  AL | 6088 | 80 | 1.3 | 0.991 (0.974 - 1.008) | 0.301 |  |  |  |  |  |
| AS-AQ:FDC | 1671 | 132 | 7.9 | 1.010 (0.991 - 1.029) | 0.322 |  |  |  |  |  |
| AS-MQ | 2216 | 11 | 0.5 | 1.031 (0.925 - 1.149) | 0.583 |  |  |  |  |  |
| DP | 3242 | 61 | 1.9 | 0.970 (0.945 - 0.997) | 0.029 |  |  |  |  |  |
| Underdose  All | 13374 | 284 | 2.1 | 1.139 (0.689 - 1.882) | 0.613 | 1567 | 1285 | 82.0 | 0.879 (0.711 - 1.086) | 0.232 |
| AL | 6088 | 80 | 1.3 | 1.333 (0.382 - 4.652) | 0.653 |  |  |  |  |  |
| AS-AQ:FDC | 1671 | 132 | 7.9 | ND |  |  |  |  |  |  |
| AS-MQ | 2373 | 11 | 0.5 | ND |  |  |  |  |  |  |
| DP | 3242 | 61 | 1.9 | 1.298 (0.729 - 2.311) | 0.375 |  |  |  |  |  |
| Low Dose (< 8mg/kg)  All | 13374 | 284 | 2.1 | 1.906 (1.065 - 3.410) | 0.030 | 1567 | 1285 | 82.0 | 1.050 (0.873 - 1.262) | 0.606 |
| AL | 6088 | 80 | 1.3 | 1.199 (0.136 - 10.597) | 0.871 |  |  |  |  |  |
| AS-AQ:FDC | 1671 | 132 | 7.9 | 1.928 (0.469 - 7.918) | 0.362 |  |  |  |  |  |
| AS-MQ | 2373 | 11 | 0.5 | ND |  |  |  |  |  |  |
| DP | 3242 | 61 | 1.9 | 2.778 (1.178 - 6.551) | 0.020 |  |  |  |  |  |
