# Supplementary material for: Gametocyte carriage in uncomplicated Plasmodium falciparum malaria following treatment with artemisinin combination therapy: a systematic review and meta-analysis of individual patient data
Source: BMC Med. 2016 May 24;14:79. doi: 10.1186/s12916-016-0621-7 (PMC4879753; doi:10.1186/s12916-016-0621-7)
Supplement: Additional file 5: Table S4. — Factors associated with the clearance of gametocytaemia after enrolment in individuals who were gametocytaemic before treatment with artemisinin combination therapy. Nobs, Number of observations; N cleared, Number of patients with day of clearance of gametocytaemia recorded. Derived haemoglobin, conversion from haematocrit: haemoglobin = (haematocrit – 5.62)/2.60 [40]; Anaemia, haemoglobin < 10 g/dL; Fever, temperature > 37.5 °C; Hyperparasitaemia, parasitaemia > 100,000 parasites per μL; weight-for-age z-score, calculated using “igrowup” package developed by WHO [41] in children < 5 years of age; Underweight, weight-for-age z-scores < –2. Proportional hazards assumption not satisfied for transmission intensity areas, Region, and artemisinin combination therapy. (DOC 59 kb) [file 12916_2016_621_MOESM5_ESM.doc]

**Supplementary Table S4. Factors associated with the clearance of gametocytaemia after enrolment in individuals who were gametocytaemic** before treatment with artemisinin combination therapy.

| Parameter | Nobs | N cleared | % | HR (95%CI) | P-value |
| --- | --- | --- | --- | --- | --- |
| ACT AS-MQ | 276 | 224 | 81.2 | 1.260 (0.996 - 1.595) | 0.054 |
| DP | 575 | 454 | 79.0 | 0.743 (0.625 - 0.882) | 0.001 |
| AS-AQ: FDC | 264 | 223 | 84.5 | 0.804 (0.634 - 1.020) | 0.072 |
| AL | 498 | 415 | 83.3 | Reference |  |
| Age < 1 year | 77 | 67 | 87.0 | 1.049 (0.764 - 1.440) | 0.768 |
| 1-4 years | 779 | 673 | 86.4 | 1.072 (0.882 - 1.304) | 0.483 |
| 5-11 years | 317 | 245 | 77.3 | 1.159 (0.965 - 1.392) | 0.114 |
| 12+ years | 439 | 331 | 75.4 | Reference |  |
| Age (years) | 1612 | 1316 | 81.6 | 0.998 (0.991 - 1.004) | 0.469 |
| Derived Haemoglobin (g/dl) | 1297 | 1094 | 84.3 | 1.010 (0.978 - 1.043) | 0.547 |
| Anemia Yes | 745 | 643 | 86.3 | 0.992 (0.867 - 1.136) | 0.912 |
| No | 552 | 451 | 81.7 | Reference |  |
| Fever Yes | 639 | 529 | 82.8 | 1.064 (0.943 - 1.201) | 0.317 |
| No | 869 | 693 | 79.8 | Reference |  |
| Sex Female | 722 | 597 | 82.7 | 1.023 (0.914 - 1.146) | 0.693 |
| Male | 888 | 719 | 81.0 | Reference |  |
| Hyperparasitaemia Yes | 46 | 36 | 78.3 | 1.157 (0.816 - 1.640) | 0.412 |
| No | 1566 | 1279 | 81.7 | Reference |  |
| Log_10_ parasitaemia | 1605 | 1308 | 81.5 | 1.023 (0.934 - 1.119) | 0.627 |
| Weight-for-age score | 833 | 723 | 86.8 | 1.054 (0.990 - 1.123) | 0.100 |
| Underweight Yes | 201 | 163 | 81.1 | 0.940 (0.778 - 1.135) | 0.519 |
| No | 632 | 560 | 88.6 | Reference |  |
| Region: Asia | 582 | 418 | 71.8 | 0.811 (0.613 - 1.071) | 0.140 |
| South America | 80 | 78 | 97.5 | 1.400 (0.662 - 2.961) | 0.379 |
| Africa | 951 | 820 | 86.2 | Reference |  |
| TIA: Low | 608 | 496 | 81.6 | 1.042 (0.749 - 1.449) | 0.806 |
| Moderate | 594 | 459 | 77.3 | 0.906 (0.653 - 1.258) | 0.557 |
| High | 396 | 347 | 87.6 | Reference |  |
