# Supplementary material for: Gametocyte carriage in uncomplicated Plasmodium falciparum malaria following treatment with artemisinin combination therapy: a systematic review and meta-analysis of individual patient data
Source: BMC Med. 2016 May 24;14:79. doi: 10.1186/s12916-016-0621-7 (PMC4879753; doi:10.1186/s12916-016-0621-7)
Supplement: Additional file 6: Table S5. — Risk of bias in individual studies included in the analysis. ACT, Artemisinin combination therapy. 1 For trials with non-ACTs, data were only analysed for gametocytaemia on enrolment and regimens, arms, randomization, concealment of treatment, sequence generation and treatment blinding are given as not applicable (NA). 2 Includes exclusions due to study design (i.e. travellers, repeated episodes). 3 Evaluated in all patients except for exclusions due to study design or protocol violations. 4 Evaluated on all included patients treated with ACT and without gametocytaemia on enrolment. 5 Proportion of patients with time to gametocyte data available but incomplete day 28 follow-up. 6 Evaluated on all included patients with gametocytaemia on enrolment treated with ACT. 7 The sensitivity of microscopy methods was classified into one of four categories: 1 = studies in which slides were specifically read for gametocytes, reviewing at least 100 microscopic high power fields or against ≥ 1000 white blood cells (WBC); 2 = microscopists specifically instructed to record gametocytes but slides were primarily read for asexual parasites ; ≥ 100 microscopic high power fields per ≥1000 WBC were read; 3 = microscopists were specifically instructed to record gametocytes; 50–99 microscopic high power fields per 500–999 WBC were read; 4 = microscopists were not specifically instructed to record gametocytes or the number of examined high power fields was < 50 or the number of WBC was < 500. 8No data, no patients with sufficient gametocyte follow-up data that could be included in the analysis. (PDF 408 kb) [file 12916_2016_621_MOESM6_ESM.pdf]

Supplementary Table S5.

study id	ACT	Study design Arm[1]	Randomisation[1]	Randomization Concealment of Treatment[1]	Sequence Generation[1]	Treatment Blinding[1]	Treatment Supervision	Protocol exclusions[2]	Missing covariates age	weight	fever	haemoglobin /haematocrit	Missing outcome on enrollment[3]	time to gametocytaemia	time to gametocytaemia[5]	time to clearance[6]	Assessment of gametocytaemia[7]
study identifier	involving ASAQ, ASMQ, AL or DP	number of arms															
ADXZX	Yes	Single	NA	NA	NA	NA	Supervised	0%	0%	0%	3%	0%	0%	0%	100%	0%	3
AJXCU	Yes	Multiple	Yes	Sealed envelopes	Computer generated	Open label	Supervised	0%	0%	0%	0%	1%	0%	3%	96%		4
BJUNF	Yes	Multiple	Yes	Sealed envelopes	Blocks of 12	Open label	Supervised	0%	0%	0%	0%	0%	0%	No data[8]	NA	No data	4
BKTSC	Yes	Multiple	Yes	Sealed envelopes	Computer generated, blocks of 12	Neither patients nor clinici	Supervised	0%	0%	0%	0%	0%	0%	No data	NA	No data	3
BMQYT	Yes	Multiple	Yes	Not mentioned	Blocks of 12	Single-blinded	Supervised	0%	0%	0%	0%	0%	0%				4
BQSYZ	Yes	Multiple	Yes	Not mentioned	Not mentioned	Open label	Supervised	0%	0%	0%	0%	8%	0%	3%	95%	0%	Not Available
BSCRC	No	NA	NA	NA	NA	NA	NA	3%	0%	0%	0%	19%	0%	NA	NA	NA	3
BSXFP	Yes	Multiple	Yes	Sealed envelopes	Computer generated	Open label	Supervised	0%	0%	0%	0%	0%	0%	No data	NA	No data	3
CCEPC	Yes	Multiple	Yes	Treatment allocation was con	Computer generated, blocks	Not applicable	Supervised/Unsupervised	0%	0%	0%	0%	0%	0%	1%	92%	0%	2
CCXPJ	Yes	Multiple	Yes	Not mentioned	Not mentioned	Single-blinded	Supervised	1%	0%	0%	0%	100%	0%	No data	NA	No data	4
CDCMJ	Yes	Multiple	Yes	Treatment allocation was con	Drawing envelope from the	Open label	Partially supervised	0%	0%	0%	100%	0%	0%	0%	91%	0%	3
CFFZN	Yes	Multiple	Yes	Not mentioned	Not mentioned	Not mentioned	Supervised	1%	0%	0%	1%	8%	0%	10%	38%	0%	3
CUDNY	Yes	Multiple	Yes	Sealed envelopes	Computer generated	Open label	Supervised	3%	0%	100%	100%	100%	0%	99%	1%	No data	4
CXJYT	Yes	Single	NA	NA	NA	NA	Supervised	0%	0%	0%	2%	100%	0%	1%	0%	No data	2
DADPZ	Yes	Multiple	Yes	Sealed envelopes	Computer generated	Open label	Supervised/Partially supervis	0%	0%	0%	0%	0%	0%	3%	97%	No data	4
DAXCM	Yes	Multiple	Yes	Not mentioned	Computer generated	Patients were treatment b	Supervised	0%	0%	1%	0%	100%	10%	9%	4%	48%	1
DBCXT	Yes	Multiple	Yes	Not mentioned	Not mentioned	Single-blinded	Supervised	71%	0%	100%	0%	0%	0%	2%	2%	No data	4
DFTUS	Yes	Multiple	Yes	Not mentioned	Not mentioned	Not mentioned	Supervised	0%	0%	1%	0%	3%	24%	3%	93%	0%	Not available
DPZDY	No	NA	NA	NA	NA	NA	NA	3%	0%	0%	0%	0%	0%	NA	NA	NA	2
DYFKY	Yes	Multiple	Randomized	Sealed envelopes	Independent statistician, blo	Open label	Supervised	0%	0%	0%	1%	0%	0%	9%	82%	0%	4
EDBXP	Yes	Multiple	Yes	Treatment allocation was con	Blocks of 15	Open label	Supervised	0%	0%	1%	0%	0%	0%	31%	63%	38%	2
EDPJN	Yes	Multiple	Yes	Randomisation lists were kept	Computer generated	Investigator blinded	Supervised	0%	0%	0%	0%	100%	0%	No data	NA	No data	3
EFTTU	Yes	Multiple	Yes	Not mentioned	Blocks of 6	Double blind	Supervised	1%	0%	0%	3%	100%	0%	3%	63%	13%	2
EGYMA	Yes	Multiple	Yes	Sealed envelopes	Block randomization	Open label	Supervised	62%	0%	0%	0%	100%	0%	3%	94%	6%	3
EKDTF	No	NA	NA	NA	NA	NA	NA	8%	0%	0%	0%	0%	1%	NA	NA	NA	Not available
EMJMA	Yes	Multiple	Yes	Sealed envelopes	Blocks of 12	Open label	Supervised	1%	0%	0%	0%	5%	0%	4%	66%	5%	3
EPDUY	Yes	Multiple	Yes	Sealed envelopes	Computer generated, blocks	The investigators recruitin	Supervised	0%	0%	0%	0%	3%	0%	No data	No data	No data	4
ESGDS	No	NA	NA	NA	NA	NA	NA	64%	0%	0%	0%	3%	0%	NA	NA	NA	3
ETFZC	Yes	Multiple	Yes	Not mentioned	Computer generated	Blinded	Supervised	0%	0%	0%	0%	0%	0%	No data	NA	No data	4
EZZJD	No	NA	NA	NA	NA	NA	NA	0%	0%	4%	0%	100%	3%	NA	NA	NA	2
FAJXQ	Yes	Multiple	Yes	Sealed envelopes	predetermined randomizatio	Open label	Supervised	0%	0%	0%	0%	0%	0%	1%	96%	10%	4
FARTM	No	NA	NA	NA	NA	NA	NA	6%	0%	0%	0%	3%	70%	NA	NA	NA	3
FBDEZ	No	NA	NA	NA	NA	NA	NA	0%	0%	0%	0%	100%	0%	NA	NA	NA	4
FEDZY	Yes	Multiple	Yes	Sealed envelopes	Computer generated, blocks	Open label	Supervised	1%	0%	0%	0%	0%	0%	4%	1%	75%	3
FFNAU	Yes	Multiple	Yes	Sealed envelopes	Blocks of 10	Open label	Supervised	0%	0%	0%	0%	0%	0%	1%	91%	1%	2
FHZMF	Yes	Multiple	Yes	Sealed envelopes	Computer generated, blocks	both patient and investiga	Supervised	2%	0%	0%	0%	57%	0%	No data	NA	No data	2
FMNNB	Yes	Multiple	Yes	Sealed envelopes	Blocks of 4	Open label	Supervised	0%	0%	0%	0%	2%	0%	5%	69%	0%	3
FNMDP	Yes	Multiple	Not mentioned	Not mentioned	Not mentioned	Not mentioned	Not mentioned	0%	0%	0%	0%	0%	0%	No data	NA	No data	4
FXFTC	Yes	Multiple	Yes	Not mentioned	Computer generated	Blinded	Supervised	0%	0%	0%	0%	0%	0%	No data	NA	No data	4
GHNKU	Yes	Multiple	Yes	Not mentioned	Not mentioned	Open label	Supervised	2%	0%	100%	56%	100%	0%	1%	89%	5%	2
GHYRA	No	NA	NA	NA	NA	NA	NA	2%	0%	2%	0%	2%	1%	NA	NA	NA	Not available
GPXJK	Yes	Multiple	Yes	Sealed envelopes	Randomised list	Open label	Supervised	0%	0%	0%	0%	0%	0%	4%	87%	4%	4
GQBEK	Yes	Single	NA	NA	NA	NA	Supervised	1%	0%	0%	0%	7%	0%	3%	44%	22%	2
GTEFH	Yes	Multiple	Yes	Cards	Drawing from box, blocks of	Not mentioned	Supervised	0%	0%	1%	1%	1%	0%	1%	95%		4
GZQDA	Yes	Multiple	Yes	Sealed envelopes	Computer generated, blocks	Open label	Supervised/Unsupervised	0%	0%	0%	0%	3%	0%	2%	92%		2
HCEMT	Yes	Multiple	Yes	Not mentioned	Not mentioned	Open label	Supervised	0%	0%	0%	1%	100%	0%	1%	1%	8%	1
HCGRD	Yes	Multiple	NA	NA	NA	NA	NA	26%	0%	1%	0%	0%	0%	3%	97%	6%	2
HJNDX	Yes	Multiple	Not mentioned	Not mentioned	Not mentioned	Not mentioned	Supervised	0%	0%	0%	0%	0%	0%	2%	95%		Not available
HMPBZ	Yes	Multiple	Not mentioned	Not mentioned	Not mentioned	Not mentioned	Partially supervised	1%	0%	100%	0%	7%	0%				Not available
JGGNM	Yes	Multiple	Yes	Sealed envelopes	Independent off site contract research organisation, stratified by country	Open label	Supervised	1%	0%	0%	0%	3%	0%	2%	92%	5%	2
JNCQS	No	NA	NA	NA	NA	NA	NA	1%	0%	0%	0%	0%	0%	NA	NA	NA	4
JRBRK	Yes	Multiple	NA	NA	NA	NA	Supervised	1%	0%	0%	0%	4%	0%				3
JTXEY	Yes	Multiple	Yes	Sealed envelopes	Computer generated, blocks	Open label	Unupervised	4%	0%	8%	0%	0%	0%	7%	10%		4
JUTXU	No	NA	NA	NA	NA	NA	NA	6%	0%	0%	0%	41%	0%	NA	NA	NA	3
JYDHH	No	NA	NA	NA	NA	NA	NA	0%	0%	100%	0%	0%	0%	NA	NA	NA	3
JZCKF	Yes	Single	NA	NA	NA	NA	Not mentioned	5%	0%	0%	0%	0%	0%	0%	98%	0%	2
KAXED	No	NA	NA	NA	NA	NA	NA	0%	0%	0%	0%	1%	0%	NA	NA	NA	3
KDDBK	No	NA	NA	NA	NA	NA	NA	48%	0%	0%	0%	0%	0%	NA	NA	NA	3
KJGJT	Yes	Multiple	Yes	Sealed envelopes	Computer generated, blocks	Open label	Supervised	1%	0%	0%	0%	2%	0%	11%	86%	16%	2
KRBXE	Yes	Multiple	Yes	Sealed envelopes	Patient selects envelope fro	Open label	Supervised/Unsupervised	0%	0%	0%	0%	100%	0%	1%	5%	47%	Not available
KTKAB	Yes	Multiple	Yes	Sealed envelopes	External statistician, blocks c	Only clinic staff was aware	Supervised	3%	0%	0%	0%	1%	1%	7%	4%	43%	2
KUCMZ	No	NA	NA	NA	NA	NA	NA	56%	0%	0%	2%	12%	0%	NA	NA	NA	3
KZBZT	Yes	Multiple	Yes	Sealed envelopes	Computer generated	Not mentioned	Supervised	0%	0%	0%	100%	100%	0%	4%	3%	.	3
MCZHT	Yes	Multiple	Yes	Sealed envelopes	Ccomputer generated	Only the study nurse was a	Supervised	0%	0%	0%	0%	0%	0%	1%	98%	0%	3
MEFSC	Yes	Multiple	Yes	Randomization was done at th	Provided by GlaxoSmithKline	Investigators, technicians p	Supervised	1%	0%	0%	0%	100%	0%	1%	2%	0%	3
MGEXQ	Yes	Multiple	Yes	Sealed envelopes	Provided by Sanofi	Double-blinded	Supervised	0%	2%	1%	1%	1%	1%	No data	NA	No data	4
MRGRH	No	NA	NA	NA	NA	NA	NA	62%	0%	1%	88%	3%	0%	NA	NA	NA	3
MTZZP	Yes	Multiple	Yes	Sealed envelopes	Blocks of 10	Open label	Supervised	0%	0%	0%	0%	0%	0%	No data	NA	No data	4
MZBCX	Yes	Multiple	Not mentioned	Not mentioned	The two therapies was alloca	Open label	Supervised	1%	0%	0%	0%	0%	0%	No data	NA	No data	4
NBSAE	Yes	Multiple	Yes	Sealed envelopes	Computer generated, blocks	Although this was an open	Supervised	0%	0%	0%	0%	0%	0%	1%	97%	0%	4
NJUCK	Yes	Multiple	Yes	Not mentioned	Not mentioned	Not mentioned	Supervised	8%	0%	0%	0%	0%	0%	3%	90%	17%	Not available
NMMSB	No	NA	NA	NA	NA	NA	NA	0%	0%	0%	0%	0%	0%	NA	NA	NA	Not available
NRXTM	No	NA	NA	NA	NA	NA	NA	1%	0%	0%	0%	0%	0%	NA	NA	NA	2
PCGTT	No	NA	NA	NA	NA	NA	NA	6%	0%	0%	0%	40%	0%	NA	NA	NA	3
PEADD	No	NA	NA	NA	NA	NA	NA	1%	0%	0%	3%	3%	3%	NA	NA	NA	2
PKGFP	Yes	Multiple	Yes	Not mentioned	Not mentioned	Not mentioned	Supervised/Partially supervis	0%	0%	0%	0%	0%	0%	1%	95%		4
PKSCU	No	NA	NA	NA	NA	NA	NA	0%	0%	100%	0%	0%	0%	NA	NA	NA	Not available
PNUNE	Yes	single	NA	NA	NA	NA	Supervised	18%	0%	0%	0%	3%	0%	0%	95%	0%	3
PUEKP	Yes	Multiple	Yes	Not mentioned	Computer generated	Open label	Supervised	0%	0%	0%	0%	1%	0%	4%	0%	12%	3
PXPZK	Yes	Multiple	Yes	Sealed envelopes	Computer generated	Although this was an open	Supervised	0%	0%	0%	0%	1%	0%	1%	98%	9%	4
QBPQM	No	NA	NA	NA	NA	NA	NA	5%	0%	0%	0%	0%	4%	NA	NA	NA	Not available
QCKHC	Yes	Multiple	Yes	Sealed envelopes	Independent statistician, blo	Open label	Supervised	36%	0%	1%	0%	0%	0%	9%	68%	46%	2
QFPXT	Yes	Multiple	Yes	Not mentioned	Computer generated	Blinded	Supervised	1%	0%	0%	0%	0%	1%	No data	NA	No data	4
QRBRC	Yes	Multiple	Yes	Sealed envelopes	Computer generated, blocks	Open label	Supervised	1%	0%	0%	0%	0%	1%	45%	47%	.	4
QZJGM	Yes	Multiple	Yes	Sealed envelopes	Independent off site contract research organization	Blinded	Supervised	3%	0%	0%	1%	1%	0%	1%	92%	0%	4
QZMAG	Yes	Multiple	Yes	Sealed envelopes	Computer generated, blocks	Open label	Supervised	0%	0%	0%	0%	0%	0%	0%	100%	0%	2
RCBGY	Yes	Multiple	Yes	Sealed envelopes	Independent statistician, blo	Open label	Supervised/Partially supervis	29%	0%	5%	5%	1%	5%	6%	48%	30%	2
RDBXS	Yes	Single	NA	NA	NA	NA	Not mentioned	0%	0%	0%	0%	0%	0%	3%	94%	5%	4
REAJS	Yes	Multiple	Yes	Not mentioned	Not mentioned	Open label	Supervised	15%	0%	0%	0%	100%	0%	1%	2%	11%	Not availble
RGDRP	Yes	Multiple	Yes	Not mentioned	Computer generated	Blinded	Supervised	0%	0%	0%	0%	0%	0%	No data	NA	No data	4
RHXNJ	No	NA	NA	NA	NA	NA	NA	6%	0%	0%	0%	0%	1%	NA	NA	NA	2
RNZFN	Yes	Multiple	Yes	Not mentioned	Blocks of 10	Open label	Supervised	1%	0%	0%	0%	0%	0%	0%	100%	0%	4
RZENT	No	NA	NA	NA	NA	NA	NA	0%	0%	0%	2%	2%	0%	NA	NA	NA	2
SATNJ	Yes	Multiple	Yes	Sealed envelopes	Computer generated, blocks	Open label	Supervised	0%	0%	0%	0%	100%	0%	0%	100%	0%	Not available
SBCEE	Yes	Multiple	Yes	Sealed envelopes	Computer generated,blocks	Open label	Supervised	93%	1%	0%	0%	0%	0%	10%	89%	8%	Not available
SEFTB	Yes	Multiple	Yes	Without concealment	Blocks of 20	Open label	Supervised	0%	0%	0%	0%	0%	0%	No data	NA	No data	4
SRDFP	No	NA	NA	NA	NA	NA	NA	2%	0%	100%	2%	0%	0%	NA	NA	NA	2
SYFQT	Yes	Multiple	Yes	Not mentioned	Not mentioned	Not mentioned	Partially supervised	0%	0%	0%	0%	0%	0%	0%	100%		3
SZRDK	Yes	Multiple	yes	Sealed envelopes	Computer generated, blocks	Open label	Supervised	1%	0%	0%	0%	3%	0%	5%	2%	33%	3
TDFKY	Yes	Multiple	Yes	The randomization codes wer	Computer generated	Single-blinded	Supervised	0%	0%	0%	0%	0%	0%	0%	98%	0%	4
TETAJ	No	NA	NA	NA	NA	NA	NA	0%	0%	0%	0%	9%	0%	NA	NA	NA	2
TJRQU	No	NA	NA	NA	NA	NA	NA	1%	0%	0%	0%	7%	0%	NA	NA	NA	3
TYKSC	Yes	Multiple	Yes	Not mentioned	Stratified by age using separ	Investigator blinded	Supervised	0%	0%	0%	0%	100%	0%	3%	92%	3%	4
UBTXH	Yes	Multiple	Yes	Sealed envelopes	Computer generated, blocks	Open label	Supervised	0%	0%	0%	0%	0%	0%	89%	11%		3
UEGEJ	No	NA	NA	NA	NA	NA	NA	1%	1%	2%	2%	4%	2%	NA	NA	NA	1
UGPAG	Yes	Multiple	Yes	Not mentioned	Not mentioned	Open label	Supervised	2%	0%	0%	0%	1%	3%	0%	10%	18%	1
UHUBT	Yes	Single	NA	NA	NA	NA	Supervised	4%	0%	1%	0%	0%	0%	5%	0%	0%	Not available
XEDNN	Yes	Multiple	Yes	Sealed envelopes	Computer generated	Open label	Supervised	1%	1%	28%	0%	2%	0%	3%	92%	50%	4
XEKED	Yes	Multiple	Yes	Not mentioned	Not mentioned	Open label	Supervised	1%	0%	1%	1%	5%	0%	3%	95%		4
XSGGK	No	NA	NA	NA	NA	NA	NA	1%	0%	0%	2%	46%	0%	NA	NA	NA	Not available
XTGNB	Yes	Single	NA	NA	NA	NA	Partially supervised	0%	0%	0%	0%	0%	0%	No data	NA	No data	4
XXFCZ	Yes	Single	NA	NA	NA	NA	Unsupervised	1%	0%	0%	0%	100%	0%	0%	0%		2
YGNQS	Yes	Multiple	Not mentioned	Not mentioned	Not mentioned	Not mentioned	Not mentioned	0%	1%	0%	0%	100%	1%	3%	89%		4
YGTAH	Yes	Multiple	Yes	The randomization list was se	Computer generated, age st	Only the study nurse was a	Supervised	0%	0%	0%	0%	0%	0%	0%	99%		3
YJXJA	No	NA	NA	NA	NA	NA	NA	0%	0%	100%	0%	20%	0%	NA	NA	NA	Not available
YKTFY	Yes	Multiple	Yes	Sealed envelopes	Not mentioned	Open label	Supervised	1%	0%	0%	18%	100%	0%	3%	69%	29%	3
YRRSK	Yes	Multiple	Yes	Not mentioned	Not mentioned	Not mentioned	Supervised	0%	0%	0%	0%	0%	0%	7%	88%		4
YYDSM	Yes	Multiple	Yes	Not mentioned	Computer generated, blocks	Open label	Partialy supervised	5%	0%	0%	0%	2%	11%	6%	4%		4
YYMQJ	No	NA	NA	NA	NA	NA	NA	0%	0%	0%	0%	0%	0%	NA	NA	NA	3
ZQSFC	Yes	Single	NA	NA	NA	NA	Not mentioned	72%	0%	0%	0%	100%	0%	3%	3%		Not available
ZYBXE	Yes	Multiple	Yes	Not mentioned	Not mentioned	Not mentioned	Supervised	0%	0%	0%	0%	0%	0%	0%	97%	0%	4
