# Supplementary material for: Gametocyte carriage in uncomplicated Plasmodium falciparum malaria following treatment with artemisinin combination therapy: a systematic review and meta-analysis of individual patient data
Source: BMC Med. 2016 May 24;14:79. doi: 10.1186/s12916-016-0621-7 (PMC4879753; doi:10.1186/s12916-016-0621-7)
Supplement: Additional file 7: Table S6. — Factors associated with the development of gametocytaemia after enrolment in individuals who were gametocyte-free before treatment with artemisinin combination therapy. Cox regression model for time to gametocytaemia. Only patients with complete 28-day follow-up are included. (DOC 35 kb) [file 12916_2016_621_MOESM7_ESM.doc]

**Supplementary Table S6. Factors associated with the development of gametocytaemia after enrolment in individuals who were non-gametocytaemic before treatment with artemisinin combination therapy.** Cox regression model for time to gametocytaemia. Only patients with complete 28-days follow-up are included. Nobs = number of patients included in the analysis; Npos = number of patients with gametocytaemia during follow-up

| Parameter | Nobs | Npos | % | HR (95%CI) | P-value |
| --- | --- | --- | --- | --- | --- |
| **Multivariable Model** | 10646 | 291 | 2.7 |  |  |
| ACT: AS-MQ | 1580 | 11 | 0.7 | 0.555 ( 0.206 - 1.494) | 0.244 |
| DP | 3023 | 61 | 2.0 | 1.756 (1.063 – 2.902) | 0.028 |
| AS-AQ: FDC | 1387 | 139 | 10.0 | 3.023 (1.745 – 5.238) | <0.001 |
| AL | 4656 | 80 | 1.7 | Reference |  |
| Age < 1 year | 565 | 22 | 3.9 | 1.021 ( 0.442 – 2.360) | 0.960 |
| 1-4 years | 5348 | 214 | 4.0 | 1.464 (0.724- 2.957) | 0.288 |
| 5-11 years | 1980 | 28 | 1.4 | 1.107 (0.605 – 2.025) | 0.741 |
| 12+ years | 2753 | 27 | 1.0 | Reference |  |
| Derived Haemoglobin (g/dL) | 10646 | 291 | 2.7 | 0.849 (0.792 - 0.910) | <0.001 |
| Fever | 10646 | 291 | 2.7 | 0.677 (0.519 - 0.884) | 0.004 |
| Log_10_ parasitaemia (/µL) | 10646 | 291 | 2.7 | 0.745 (0.607 - 0.916) | 0.005 |
