# Supplementary material for: Gametocyte carriage in uncomplicated Plasmodium falciparum malaria following treatment with artemisinin combination therapy: a systematic review and meta-analysis of individual patient data
Source: BMC Med. 2016 May 24;14:79. doi: 10.1186/s12916-016-0621-7 (PMC4879753; doi:10.1186/s12916-016-0621-7)
Supplement: Additional file 8: Table S7. — Sensitivity analysis: variation in model coefficients after exclusion of individual studies. 1 Estimates as obtained in the final multivariate models and listed in main tables. 2 RSD, Relative standard deviation was calculated as a ratio of standard deviation to mean of the estimates (odds ratio or hazard ratio) calculated by fitting models with one study excluded at a time. (DOC 44 kb) [file 12916_2016_621_MOESM8_ESM.doc]

**Supplementary Table S7. Sensitivity analysis: variation in model coefficients after exclusion of individual studies**

**^1^ Estimates as obtained in the final multivariate models and listed in main tables.**

**^2^**RSD = Relative Standard Deviation was calculated as a ratio of standard deviation to mean of the estimates (OR or HR) calculated by fitting models with one study excluded at a time.

| Multivariable Model | OR (95%CI) or HR (95%CI) ^1^ | RSD (%)^2^ |
| --- | --- | --- |
| **Gametocytes on enrolment - Africa** |  |  |
| Age (years) | 0.984 (0.974 – 0.994) | 0.1 |
| Derived Haemoglobin (g/dL) | 0.788 (0.770 – 0.807) | 0.2 |
| Log_10_ Parasitaemia(/µL) | 0.617 (0.575 – 0.662) | 0.7 |
| Fever | 0.633 (0.579 – 0.691) | 0.8 |
| **Gametocytes on enrolment - Asia** |  |  |
| Age (years) | 0.988 (0.982 – 0.994) | 0.0 |
| Derived Haemoglobin (g/dL) | 0.672 (0.648 – 0.697) | 0.6 |
| Log_10_ Parasitaemia (/µL) | 0.735 (0.669-0.807) | 1.1 |
| Fever | 0.811 (0.689 – 0.954) | 1.3 |
| Sex (M) | 1.252 (1.073 – 1.462) | 1.8 |
| **Time to gametocytaemia** |  |  |
| ACT: AS-MQ | 0.566 ( 0.225 - 1.420) | 8.4 |
| DP | 2.029 (1.240 – 3.317) | 3.8 |
| AS-AQ: FDC | 4.014 (2.398 - 6.719) | 3.3 |
| AL | Reference |  |
| Age < 1 year | 1.707 ( 0.778 – 3.747) | 4.8 |
| 1-4 years | 2.303 (1.208 - 4.392) | 3.8 |
| 5-11 years | 1.418 (0.795 - 2.527) | 3.5 |
| 12+ years | Reference |  |
| Derived Haemoglobin (g/dL) | 0.828 (0.774 - 0.886) | 0.5 |
| Fever | 0.653 (0.503 - 0.848) | 2.2 |
| Log_10_ parasitaemia (/µL) | 0.757 (0.624 - 0.917) | 1.2 |
| **Time to clearance** |  |  |
| ACT: AS-MQ | 1.260 (0.996 - 1.595) | 1.8 |
| DP | 0.743 (0.625 - 0.882) | 1.3 |
| AS-AQ: FDC | 0.804 (0.634 - 1.020) | 0.8 |
| AL | Reference |  |
| Log_e_ gametocytaemia (/µL) | 0.867 (0.829 -0.907) | 0.3 |
